# An agent-based model of the effects of limited vaccination on novel respiratory infections

**DOI:** 10.1016/j.jclinepi.2026.112171

**Published:** 2026-01-24

**Authors:** Mary G. Krauland, Alexis J. Mandell, Katherine V. Williams, Richard K. Zimmerman, Mark S. Roberts

**Affiliations:** a Department of Health Policy and Management, University of Pittsburgh School of Public Health, Pittsburgh, Pennsylvania; b Public Health Dynamics Laboratory, University of Pittsburgh School of Public Health, Pittsburgh, Pennsylvania; c Department of Family Medicine, University of Pittsburgh School of Medicine, Pittsburgh, Pennsylvania

**Keywords:** Vaccination, Pandemic virus, Vaccine distribution, Respiratory virus, Agent-based modeling, Limited vaccination

## Abstract

**Objectives:**

Vaccination is a safe and effective method for preventing infectious diseases but for novel pathogens vaccine supplies may be limited. We modeled distribution of a limited vaccine supply to specific age groups to investigate the effectiveness at preventing infection and mortality of a novel influenza-like virus.

**Methods:**

We used the agent-based modeling platform Framework for Reconstructing Epidemic Dynamics. Limited supplies of vaccine were administered to target age groups. Increased vaccination rates in the 5–17 year old age group tested the ability of single-age group vaccination to interrupt viral transmission. Mortality was estimated using patterns associated with historic pandemic respiratory pathogens.

**Results:**

In all cases limited vaccination had relatively little impact on overall infections. Distribution of vaccine to 5–17 years olds stopped transmission only at very high vaccine coverage, high vaccine effectiveness and relatively low viral transmissibility. The best strategy for decreasing mortality in all modeled scenarios was to target the age group with the highest mortality.

**Conclusion:**

Results suggests that in the event of a highly transmissible disease, limited vaccine supplies will not interrupt transmission. Vaccine distribution protecting the most vulnerable from severe outcomes, even without limiting transmission, was the most effective strategy for preventing mortality.

## Plain Language Summary

In the case of an emerging disease, vaccine supplies may be limited during the time of high transmission. In such a situation, vaccinating the age group with the highest transmission rate may not be the best strategy to limit mortality, since it will not stop transmission or produce herd immunity. Vaccination may be better used to target the age group with the highest mortality, which may be different for different diseases.

## 1. Introduction

Vaccination is the most effective and most widely used preventive for seasonal influenza and other diseases spread by the respiratory route [1,2]. Such vaccines are typically produced in sufficient quantities to administer to as many people as are willing to be vaccinated in the United States, although there may be limited quantities available in some locales. In the case of an epidemic of a newly emerging respiratory infection requiring new vaccine development, that vaccine may not be available soon enough in sufficient quantity to fully vaccinate the willing population when viral transmission is widespread [3]. This raises the question of determining the best strategy for allocation of vaccine when supplies are limited. Vaccination can be administered randomly on a first-come, first-served basis or can be administered in a targeted strategy to stop transmission by vaccinating the segment of the population that has the highest transmission rates. Alternatively, the strategy may be to limit severe disease cases by vaccinating the most vulnerable members of the population. Most often in respiratory virus transmission, the highest rate of transmission is in school-aged children while the most vulnerable to severe outcomes are the very young and older age group individuals, although the pattern sometimes differs. The question of limited existing population immunity and a limited quantity of vaccine was pertinent in the COVID-19 pandemic when vaccines were first available, during the 2009 flu pandemic, when supplies were not adequate [4] and may be relevant again if H5N1 becomes highly human transmissible or if another, currently unknown, infectious agent emerges.

The impact of limited vaccination or of vaccination by age group has been seen in population studies. Decreases in influenza and influenza-related pneumonia cases in older age groups have been found to be associated with influenza vaccination in children [5–9]. Vaccination strategies have also been modeled using a variety of methods but most commonly using compartmental and statistical modeling [10–17]. Modeling studies have addressed a variety of outcomes, including reductions in infections, hospitalizations and deaths but also geographic spread and timing of vaccination. However, agent-based model (ABM) studies, which natively incorporate both indirect benefits of vaccination and herd immunity and which enable highly granular distribution of individual characteristics and transmission dynamics, are lacking. In an ABM, each agent may have personal characteristics that include their age and gender but also their own household characteristics (number and ages of other household members), their school or workplace characteristics including total size and their neighborhood characteristics, which include the density of population in the neighborhood. An agent will have their own susceptibility to infection based on whether or not they are vaccinated but will also have their own exposure network which, while not unique, is quite variable in the population. Probability of infection is a function of the calibrated contact rates for that network. Compartmental models are not able to explore disease transmission at this level of granularity. Therefore, while this topic has been explored in the past, the use of an ABM gives a much more granular and nuanced estimation of the impact of vaccination. In addition, consistency of findings across multiple modeling methodologies adds credence to those findings.

To investigate which vaccination strategy best protects the health of the public, we implemented an ABM of a respiratory infection similar in natural history to influenza but with varying levels of transmissibility and varying levels of severe outcomes, using the Framework for Reconstructing Epidemic Dynamics (FRED) ABM platform. Agents in ABMs represent individuals in the simulation population, analogous to hosts in the classic epidemiologic triad. Simulations included administration of a limited supply of vaccine to target age groups and compared the reduction in case burden and severe outcomes between the scenarios. Comparison groups included random vaccination and no vaccination. To determine the effectiveness of higher vaccine uptake in a single age group, some scenarios vaccinated the 5–17 year old group at higher rates. Our results may be useful in the event of limited overall US vaccine supply or local US state or county vaccine shortages and may inform vaccination policies in other countries.

## 2. Methods

### 2.1. The Framework for Reconstructing Epidemic Dynamics

FRED is an ABM platform that uses synthetic populations based on the 2010 US Census. FRED simulations include models for one or more conditions which may include infectious diseases. Infectious disease conditions spread through interactions between agents in mixing groups including their homes, schools, workplaces, and neighborhoods. Each condition includes a set of model states (eg, susceptible, infectious) and rules for transitions between those states. FRED has previously been described in detail [18–21]. This study used an existing modified SEIR model for influenza constructed as a state-transition model (Fig S1) but designed to simulate a strain of influenza for which there is no preexisting immunity. Agents in the model are vaccinated over a period of 6 weeks starting on September 16th. Two weeks after vaccination, agents receive the level of protection specified by the vaccine effectiveness (VE). For each of the chosen scenarios, VE was varied as a 40%, 60%, or 80% reduction in susceptibility to infection for vaccinated agents. We modeled an outbreak taking place during one typical respiratory virus season in the United States (August 1-July 31) (Fig S2). A total of 50 initial influenza infections are inserted randomly in the population on October 15th. Infections in the model are assumed to be asymptomatic at a rate of 25% of total infections [22]. Simulations were run with 100 repetitions to provide means and standard deviations for each scenario. The number of iterations provides a stable estimate but with acceptable computational complexity (Fig S3). Further model details can be found in the Supplementary Materials (Table S1).

In the FRED system the reproduction number (R_0_) is not an input. Rather it is produced as a combination of model characteristics such as transmissibility of the virus and length of infectious period, characteristics of agent interactions and characteristics of the agents themselves, such as susceptibility to infection. Simulations produce an approximate R_0_ as an output of the model. For a given disease natural history and population, appropriate case burdens are produced by calibrating a transmissibility parameter. A range of transmissibility values were used to produce a range of ~R_0_ values representative of seasonal and pandemic influenza (Table S2). Representative R_0_s were obtained from the literature [23]. Further explanation of reproduction number and transmissibility in FRED are included in the Supplementary Materials.

Simulations used a synthetic population of ~1.2 million individuals located in Allegheny County, Pennsylvania, and drawn from the 2010 US Census [24–27]. Allegheny County contains the city of Pittsburgh and the surrounding suburbs. This population is similar in demographics and household structure to the United States as a whole (Table S3). In prior work, we have tested similar models in other county locations with diverse demographics, and, while absolute number of infections differ, results were similar across locations [28].

### 2.2. Limited vaccine analysis

To determine the impact of age group–specific vaccination strategies with limited vaccine availability, transmission was modeled while randomly vaccinating agents within a single age group with all available vaccine doses (Table 1). In each model, only agents in the specified age group were vaccinated. Specified age groups were 5–17, 18–49, 50–64, and 65 and up. The vaccinated group received either 50,000 or 100,000 doses. These numbers were based on an estimate of 10 million doses for the entire United States, scaled to our simulation population, which would provide ~3% uptake overall. This estimate is in line with some estimates of possible supplies of an H5N1 vaccine [29]. The primary outcome in the single age group vaccination strategies was case burden compared to random allocation of the same limited number of vaccine doses across all age groups and to the absence of any vaccination. Percent reduction in infections compared to no vaccination was also calculated.

Deaths were estimated to determine each vaccination strategy’s impact on severe outcomes. Estimated deaths were calculated for each vaccination strategy by applying age-specific case fatality rates for the United States 2017–2018 H3N2 seasonal influenza outbreak, the H1N1pdm09 influenza pandemic, the 1918 influenza pandemic, and the first wave of the COVID-19 pandemic to the model output for symptomatic infections (Table 2) [30–34]. Further detail about the method used to derive number of deaths is included in the Supplementary Materials. These four historical pandemics or outbreaks were chosen to represent a range of severity for different age groups. H1N1pdm09 influenza and the 1918 pandemic were more deadly among younger age groups than is usual for influenza, while the severe 2017–2018 H3N2 season in the United States and the 2020–2021 phase of the COVID-19 pandemic were more deadly for older populations. Number of deaths resulting from each age group–specific vaccination strategy was compared with no vaccination and random allocation of vaccine doses.

### 2.3. Increased vaccination to interrupt transmission in the general population

To determine if transmission could be interrupted by an age group–specific vaccination strategy, an increasing proportion of the 5–17 age group were vaccinated since that age group often drives influenza transmission [35]. Coverage of the 5–17 age group was increased from 10% to 90% to determine at what level of coverage, if any, transmission of infections was interrupted (Table 1). Agents in other age groups remained unvaccinated in these scenarios. The increase in coverage was modeled across a range of ~R_0_ and VE.

## 3. Results

### 3.1. Limited vaccination

**Figure 1.**
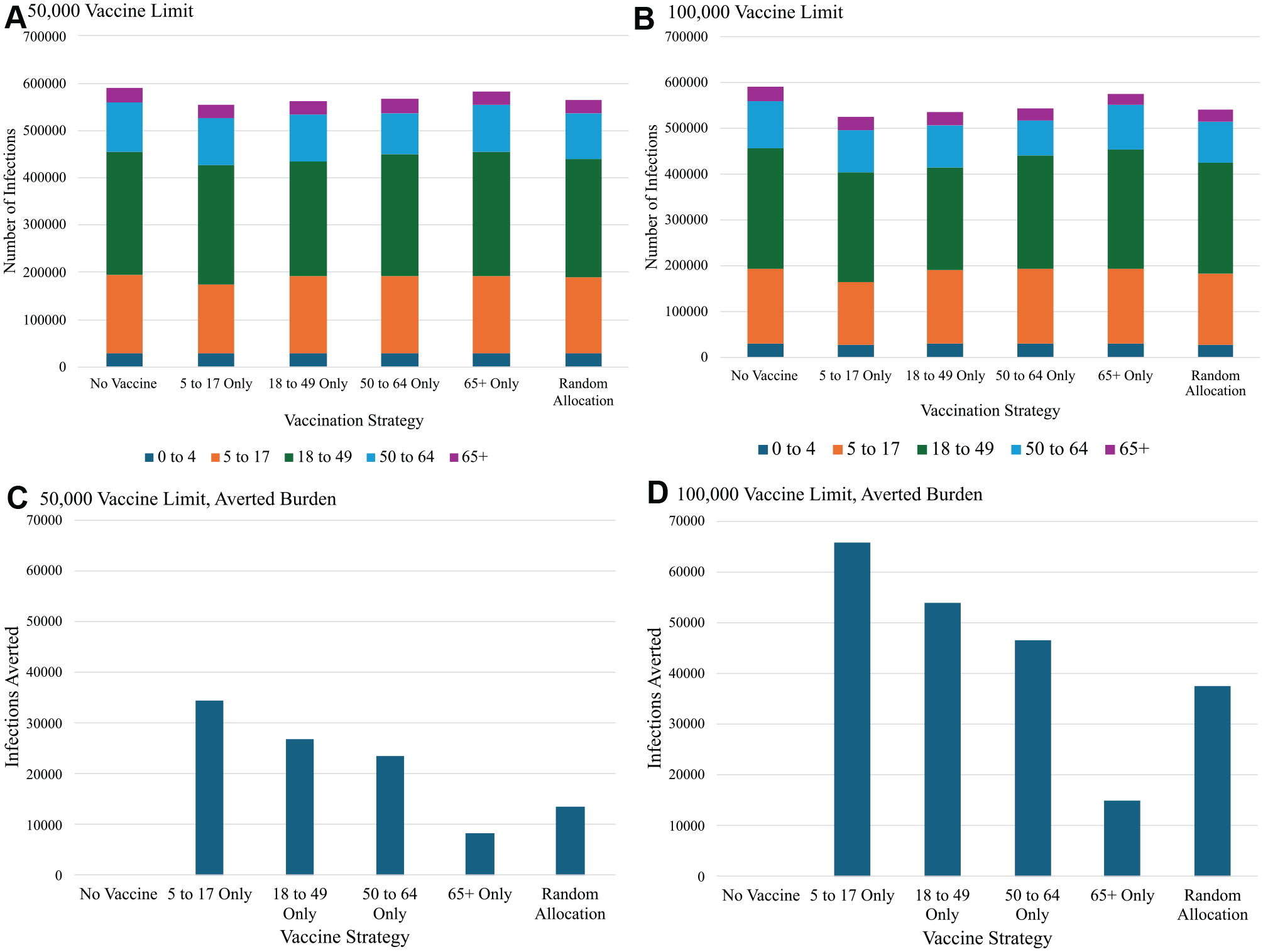
Number of infections and number of infections averted by vaccination strategy and number of vaccine doses available during the epidemic. For age group–specific strategies, all available vaccines are distributed to agents within the specified age groups and all other agents remain unvaccinated. Figures show results modeled with 60% VE and an ~R_0_ = 1.64. Case burden by age group for 50,000 vaccine doses (A) and 100,000 vaccine doses (B); total cases averted by 50,000 doses (C) and 100,000 vaccine doses (D). VE, vaccine effectiveness.

Administration of 50,000 or 100,000 vaccine doses had limited impact on the burden of infection compared to no vaccine, even when targeting high transmission groups such as children or working adults, either in total population burden or in age group–specific burden (Fig 1, Table 3, Figs S3 and S4, Tables S4 and S5, Supplementary Spreadsheet). The largest decrease in infections occurred when vaccinating the 5–17 age group only for both a 50,000 and a 100,000 vaccine limit. For example, at an ~R_0_ of 1.64, there was a reduction in cases of 6.7% or 11.1%, respectively, in the total population and reduction of 11.7% or 16.3% in the 5–17 year old age group for scenarios with 60% VE. Decreases in case burden in the 18–49 or 50–64 age group vaccination strategies were comparable to the decrease in infections with completely random vaccine allocation for 60% VE and an ~R_0_ of 1.64 (~4% or 8%-9% reduction for age group vaccination, 4% or 8% for random allocation). An analysis across higher and lower ~R_0_ values revealed that the limited vaccine has less impact as the infectiousness of the virus increases, but the pattern across age group vaccination strategies remained consistent (Figs S4 and S5). Scenarios with decreased or increased VE resulted in lower or higher impacts but the pattern of decrease in case burden due to vaccination was the same (Supplementary Spreadsheet).

### 3.2. Mortality by age group

Historical mortality patterns by age differ for H1N1 pdm09 and the 2017–2018 H3N2 seasonal influenza (Table 2) [30–32,34]. H1N1 pdm09 resulted in death rates in persons 18 to 64 that were similar to the rates in the 65 and older age group, which is atypical for influenza. The 2017–2018 H3N2 seasonal outbreak in contrast had a much higher death rate in 65 and older than in younger age groups; this pattern is more commonly seen in influenza. Additionally, mortality rates for the 2017–2018 H3N2 seasonal influenza were similar to H1N1 pdm09 in ages up to 49 but double for ages 50–64 and ~31 times higher for 65 and older. Mortality rates in the 1918 influenza pandemic were high in the 65 and over age group but also high in the 4 and under age group, and higher than usual for influenza in the 18–49 age group. Mortality for COVID-19 was highest in persons 65 and older.

**Figure 2.**
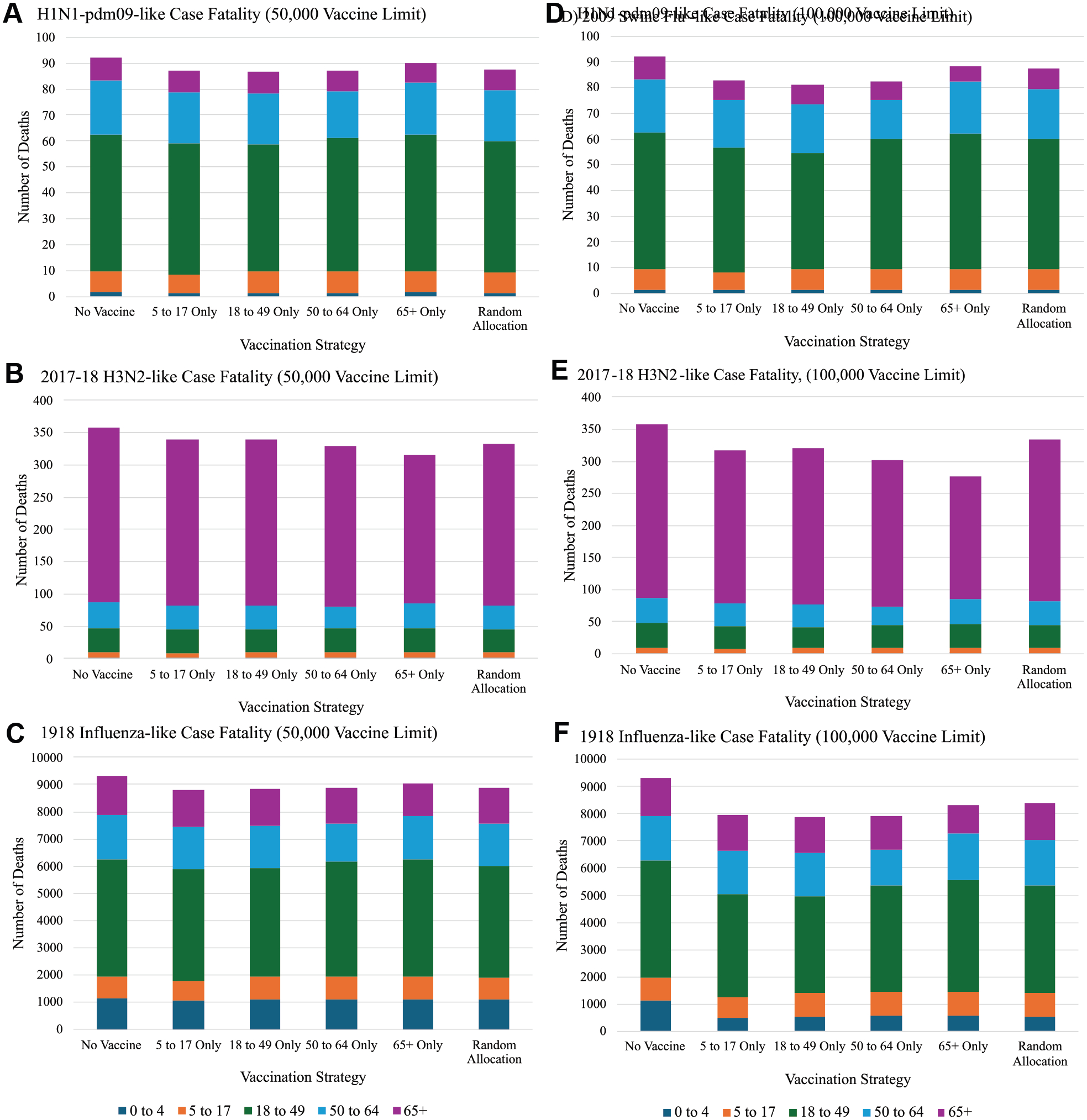
Estimated deaths using historical case-fatality rates (CFRs) by vaccination strategy and number of vaccine doses available during the epidemic (50,00 and 100,000) for simulations with 60% vaccine effectiveness and ~R_0_ = 1.64. A and B: H1N1-pdm09-like CFR, C and D: 2017–2018 H3N2-like CFR, E and F: 1918 influenza-like CFR.

The effectiveness of vaccinating single age groups at reducing estimated deaths differed when analyzed by strain type (Fig 2, Table 3, Tables S4 and S5, Figs S6–10, Supplementary Spreadsheet). For a strain with an H1N1 pdm09 mortality pattern, vaccinating 18–49 year olds only produced the largest reduction in estimated deaths at 60% VE and ~R_0_ = 1.64, but overall differences were minimal among the strategies (Fig 2, panels A and B). For a strain with an H3N2-type mortality pattern, the greatest reduction in mortality occurred when vaccinating agents aged 65 and older (Fig 2, panels C and D). Mortality followed a similar pattern when estimated using rates obtained from the COVID-19 first wave (Figs S6–10) but differed when using rates from the 1918 influenza pandemic, due to higher mortality in the 0–4 age group (Fig 2, panels E and F). As ~R_0_ increased, the impact of vaccination was attenuated but the pattern across vaccination strategies remained similar (Tables S4 and S5, Supplementary Spreadsheet, Figs S6–10).

### 3.3. Increased vaccination to interrupt transmission

**Figure 3.**
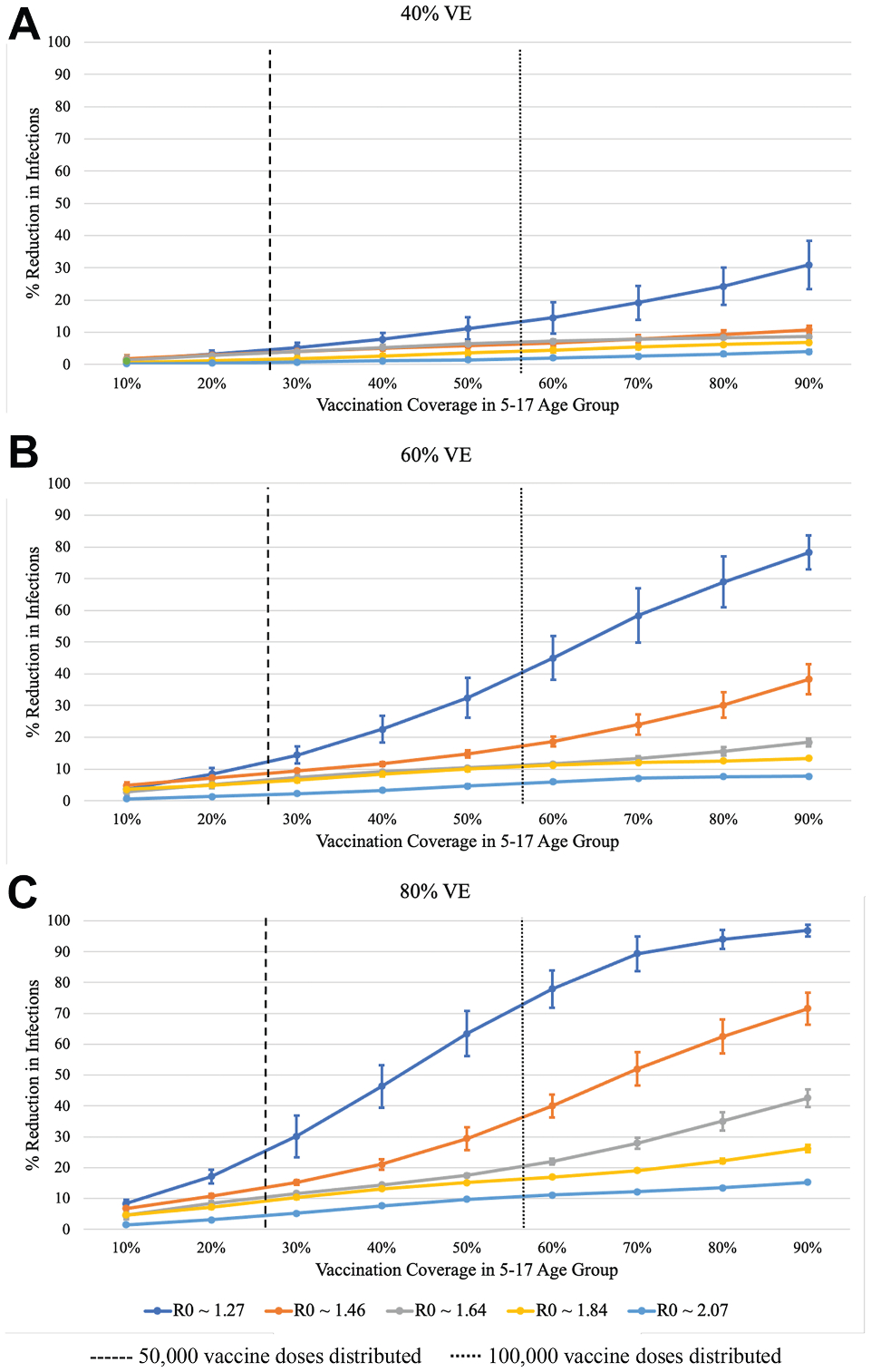
Percent reduction in infections compared to no vaccination resulting from increasing vaccination coverage in the 5–17 age group at 40% (A), 60% (B), and 80% (C) vaccine effectiveness (VE) values across a range of ~R_0_ values. X-axis: vaccination coverage; Y-axis: percentage reduction in infections compared to no vaccination. Error bars represent standard deviation.

Increased vaccine uptake in the 5–17 age group was of limited success in interrupting transmission. At low VE (40%), no level of vaccine uptake in the 5–17 year old group was sufficient to stop transmission in the general population (Fig 3A, Figs S11 and S12, Supplementary Spreadsheet) or in the 5–17 year age group for any tested value of ~R_0_. For example, at that level of VE, vaccine uptake of 90% in the 5–17 age group resulted in only 31% overall reduction in cases from the no vaccination scenario when the ~R_0_ was 1.27 and only a 4% reduction in cases at an ~R_0_ of 2.07. A vaccine with VE of 60% was more effective in models with the lowest tested ~R_0_ (1.27) but still required 90% vaccine uptake to produce a 78% reduction in transmission (Fig 3B). For ~R_0_ of 2.07, 90% vaccination resulted in only an 8% reduction in cases in the entire population. A vaccine with 80% effectiveness almost ended transmission in the lowest ~R_0_ for values of uptake ≥70% (Fig 3C). However, for higher ~R_0_ scenarios even 90% vaccine uptake failed to cause substantial reduction in transmission. At ~R_0_s of 1.93 and above, even 90% uptake in the 5–17 year old group produced ≤20% reduction in cases in the population overall.

## 4. Discussion

Considerable work has been done to investigate optimum vaccination strategies in the case of limited vaccine supply. This effort was particularly intense during the COVID-19 pandemic [11,12,14,15,17,36] and remains a question of interest for seasonal influenza, pandemic influenza, and for possible future novel pathogens. Some studies suggest that the working age population is the best target for vaccination, while others suggest that vaccinating school age children is a more effective strategy [5–9,37,38]. Lee et al [39] suggest that the best vaccination strategy when interruption of transmission is the main goal and vaccine supplies are limited is one in which 20–39 year olds are vaccinated, followed in effectiveness by vaccinating 6–12 year olds. Multiple studies, including both modeling and surveillance studies support that influenza transmission is highest in school age children and that therefore that age group is best to target when interruption of transmission is the goal [6,9,37,38,40]. The specific question we investigated was how effective a limited number of vaccine doses could be if available at the beginning of an outbreak and how that vaccine could best be targeted. The key findings of our study are that limited vaccination, whether to a target age group or randomly allocated to the population, while decreasing cases, is unlikely to interrupt transmission sufficiently to protect vulnerable subgroups and that the best strategy in a situation in which vaccine supply is limited is to devote that vaccine to the subgroup with the highest level of serious disease. Additionally, even high levels of vaccination in the highest transmission group were insufficient to halt transmission or to reach herd immunity in the population except possibly at very high levels of uptake, high VE and low ~R_0_.

The use of an ABM for this study provides greater granularity in individual contact patterns than is possible with compartmental models and allows the identification of emergent properties of a system. Vaccination in one age group affects all age groups by changing the rate of infections in all mixing groups and this may be difficult to capture in a purely equation based model. Our ABM uses a more natural method for transmission by having agent interactions spread infections. Therefore, our results add to the literature on targeted vaccination and can be useful in adding to the existing knowledge on this subject.

Vaccination of the vulnerable population will likely be the most effective method for limiting severe disease and mortality but the vaccination target groups may not always be the same. For example, the 1918 influenza pandemic resulted in higher reported mortality in the 0–4 year old age group than is commonly found in seasonal influenza or than was found in COVID-19, which would argue for vaccination of members of that age group old enough to be vaccinated along with vaccination of their close contacts. If a particular age group was both a driver of transmission and a vulnerable group, it might be optimal to target that group; that question was not part of this study and deserves further investigation. Our use of mortality rates from historical outbreaks is not meant to be predictive nor did we calibrate to deaths; the purpose was to illustrate that the same number of cases can cause vastly different mortality patterns depending on the particular disease and this will affect which is the most effective vaccination strategy.

Our results support the stockpiling of vaccines for viruses with potential to be become easily human transmissible, such as H5N1, for which a limited reserve currently exists. If H5N1 became readily transmissible, it could move quickly through the population, possibly not allowing time for vaccine production capacity to be increased. Therefore, while expensive, maintenance of a large vaccine reserve might be cost-effective.

### 4.1. Limitations

This study, by design, looked only at the situation in which a single limited number of vaccine doses was available over the course of a single season in a single locale. In the case where vaccine supply was made available on a rolling basis over time, the results may differ, depending on how many doses are available over the course of the season or infection wave, how long sustained transmission lasts and at what point in the epidemic the doses are available. This study does not include specific locations of high transmission, such as hospitals, in which different targeting strategies may be more effective but does include homes, schools, workplaces, and local area contacts, which can be expected to contribute most to viral transmission. While simulations in this study used a single county population, that population is demographically similar to the US 2020 population and therefore should be a reasonable surrogate for the country as a whole.

## Supplementary Material

supplementary material

supplementary spreadsheet

Supplementary data related to this article can be found at https://doi.org/10.1016/j.jclinepi.2026.112171.

## Figures and Tables

**Table 1.** Vaccination strategies

Strategy	Age group	Number of vaccine doses	Percent of age group vaccinated^a^	Number in age group (% of total population)
No vaccination	All ages	0	0	1,218,695 (100)
Single age group	5–17	50,000	27	186,501 (15.3)
	5–17	100,000	54	
	18–49	50,000	10	505,676 (41.5)
	18–49	100,000	20	
	50–64	50,000	19	256,540 (21.0)
	50–64	100,000	38	
	≤65	50,000	24	206,962 (17.0)
	≤65	100,000	48	
Random vaccination all ages	All ages	50,000	4	1,218,695 (100)
	All ages	100,000	8	
Increasing coverage without vaccine limit	5–17	18,650	10	186,501 (15.3)
		37,300	20	
		55,950	30	
		74,600	40	
		93,250	50	
		111,900	60	
		130,550	70	
		149,200	80	
		167,851	90	

a Number of vaccines distributed/number of agents in the age group.

**Table 2.** Literature-derived case fatality rates by age group for the H1N1 pdm09 flu pandemic, the 2017–2018 US H3N2 seasonal outbreak, the 1918 influenza pandemic, and the 2020–2021 US COVID-19 pandemic

Age group	H1N1 pdm09	2017–2018 US H3N2	1918 influenza	2020–2021 US COVID-19
0–4	0.00657%	0.00313%	5%	0%
5–17	0.00657%	0.00703%	0.68%	0.013%
18–49	0.0270%	0.0194%	2.19%	0.105%
50–64	0.0270%	0.0510%	2.3%	0.63%
65+	0.0273%	0.8561%	4.7%	6.2%

**Table 3.** Total infections for ~R_0_ 1.64 and varied vaccination strategies at 60% vaccine effectiveness with estimated mortality at rates derived from historical outbreaks, reported as mean (standard deviation)

Total infections or deaths	No vaccination	Vaccinate^a^ 5–17	Vaccinate 18–49	Vaccinate 50–64	Vaccinate ≥65	Random allocation
50,000 vaccines						
Total Infections	589,746 (6527.21)	555,361 (4887.18)	562,987 (6981.04)	566,210 (6398.17)	581,497 (6570.92)	565,008 (7458.11)
Deaths H1N1 pdm09 rates^b^	92.01 (0.86)	87.13 (0.64)	86.65 (0.92)	87.12 (0.84)	90.05 (0.86)	87.58 (0.98)
Deaths 2017–2018 H3N2 rate^c^	357.70 (2.34)	338.62 (1.75)	340.00 (2.50)	329.39 (2.29)	315.56 (2.36)	333.08 (2.67)
Deaths 1918 influenza rates^d^	9324.18 (89.93)	87,99.31 (67.33)	8840.44 (96.18)	8869.70 (88.15)	9052.71 (90.53)	8872.75 (102.76)
Deaths COVID-19 rates^e^	2669.15 (16.89)	2530.84 (12.64)	2537.49 (16.55)	2437.78 (16.55)	2360.89 (17.00)	2483.30 (19.29)
100,000 vaccines						
Total infections	589,746 (6527.21)	524,017 (2200.87)	543,195 (7007.22)	535,760 (6321.97)	574,862 (5863.59)	541,631 (6337.08)
Deaths H1N1 pdm09 rates	92.01 (0.86)	82.99 (0.29)	81.23 (0.92)	82.41 (0.83)	88.33 (0.77)	87.58 (0.83)
Deaths 2017–2018 H3N2 rate	357.70 (2.34)	316.42 (0.79)	320.66 (2.51)	301.86 (2.27)	276.06 (2.10)	333.08 (2.27)
Deaths 1918 influenza rates	9324.18 (89.93)	7936.54 (30.32)	7887.87 (96.54)	7915.37 (87.10)	8308.04 (80.79)	8403.66 (87.31)
Deaths COVID-19 rates	2669.15 (16.89)	2364.56 (5.69)	2393.52 (16.35)	2214.09 (16.35)	2071.62 (15.17)	2483.30 (16.39)

a All vaccinations were within the specified age group except for the random allocation scenario, in which vaccination was applied to all age groups.

b H1N1 pdm09 death rate by age group.

c 2017–2018 US H3N2 death rate by age group.

d 1918 Influenza death rate by age group.

e 2020–2021 US COVID-19 death rate by age group.

## Data Availability

Data will be made available on request.

## What is new?

### Key findings

- Limited vaccine doses are ineffective at stopping transmission and therefore, the best use of limited vaccine supplies is to target the age group with the highest morbidity. Limited vaccination in the highest transmission rate group can be effective if the virus has relatively low transmissibility, but in a high transmission scenario, even 90% vaccine uptake will be relatively ineffective at ending transmission.

### What this adds to what is known?

- Most existing surveillance studies were observational and only identified effects of vaccinating one age group (generally children) on other age groups (usually older adults). There are some compartmental model studies but no agent-based modeling studies that address our specific question in similar depth. Our agent-based model platform enables a more realistic and nuanced assessment of the impact of limited vaccine supply and targeted vaccination and therefore adds to the body of knowledge on this subject.

### What is the implication and what should change now?

- Limited quantities of vaccine will not be effective at stopping transmission.
- An understanding of the mortality pattern of a given virus is useful in determining the optimal target population for vaccination because the most vulnerable groups are not always the same.
- In the case of a pathogen with significant potential for development of effective human-to-human transmission, it would be prudent to maintain a significant stockpile of vaccine, if one were available. Surveillance for localized outbreaks could be useful in targeting locations for vaccine intervention, rather than distributing vaccine more broadly.
