# Supplementary material for: An agent-based model of the effects of limited vaccination on novel respiratory infections
Source: J Clin Epidemiol. Author manuscript; Available in PMC 2026 Jun 26. (PMC13308560; doi:10.1016/j.jclinepi.2026.112171)
Supplement: supplementary material [file NIHMS2184475-supplement-supplementary_material.docx]

Supplementary Materials

1. FRED model details
2. FRED transmissibility parameter
3. Allegheny County Synthetic Population
4. Mortality calculation method
5. Additional results
6. References
7. *FRED model details*

Simulations used a modified SEIR model with added states for asymptomatic infections, hospitalization, and death (Figure S1). Asymptomatic agents receive a 50% reduction in their transmissibility. Once infected, an agent’s susceptibility to future infection decreases to 0, and this protection wanes at a rate of 3% per month. Simulations ran from August 1 to July 31, to encompass a typical respiratory virus season (Figure S2). Vaccination begins on September 16^th^ and continues over a period of 6 weeks (Figure S2). Agents are chosen for vaccination randomly based on age group specific rates (5-17, 18-49, 50-64, or 65 and up) or are randomly chosen for the comparison simulations. Once an agent is chosen for vaccination, time to vaccination is drawn from a uniform distribution of 1 to 45 days. Two weeks after receiving the vaccine, vaccinated agents receive a decrease in their susceptibility equal to the specified vaccine effectiveness (VE), and vaccine protection wanes at a rate of 7% per month. Fifty initial cases are seeded on October 15^th^ of the simulation. Agents are chosen randomly from the population to be part of the initial cases of the outbreak so that they are distributed evenly around the simulation area and in all age groups. Infections are transmitted as a result of interactions in homes, schools, workplaces and neighborhoods ^1^. Model inputs are summarized in Table S1.

Simulations include a condition which has symptomatically infectious agents voluntarily quarantine at home at a rate of 40% until they recover. Quarantining agents can still infect household members but not other agents. Asymptomatic agents do not quarantine, since they either have no symptoms or are only very mildly symptomatic.

Each parameter set was run for 100 simulations. We performed a limited sensitivity analysis on the effect of number of simulations on the outcome of mean total infections and on the associated standard deviation. While an increase in number of iterations from 10 to 20 resulted in a 10% increase in total infections, increases were less for additional increments over 50 iterations (Figure S3).


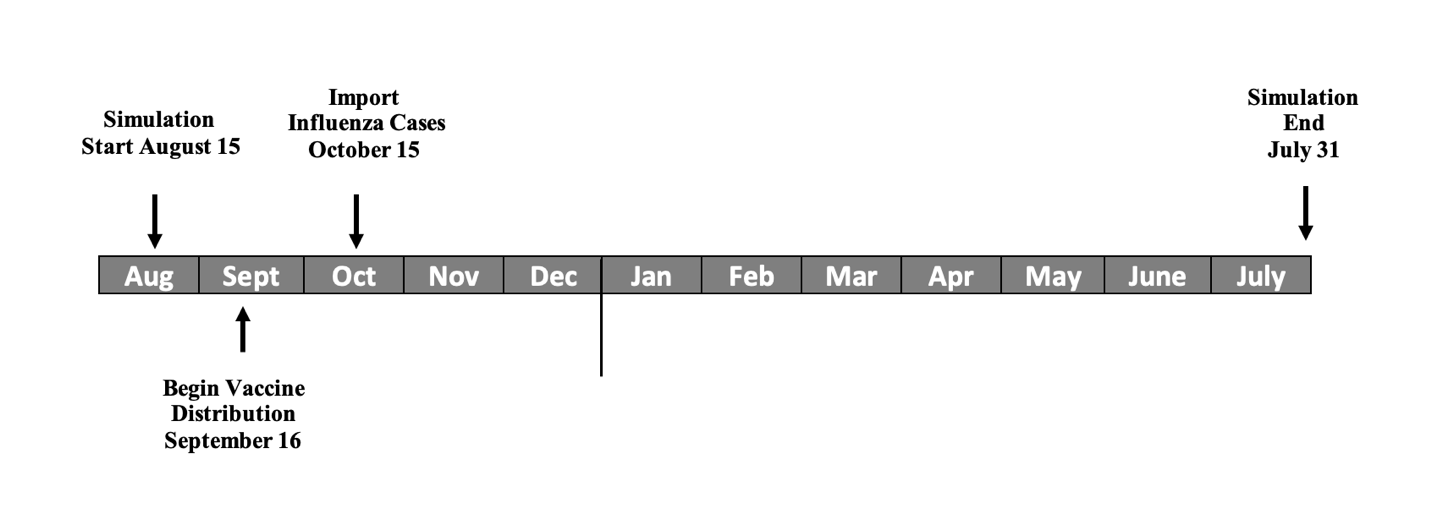


**Figure S2.** Simulation timeline.

**Figure S1.** FRED influenza model state diagram.


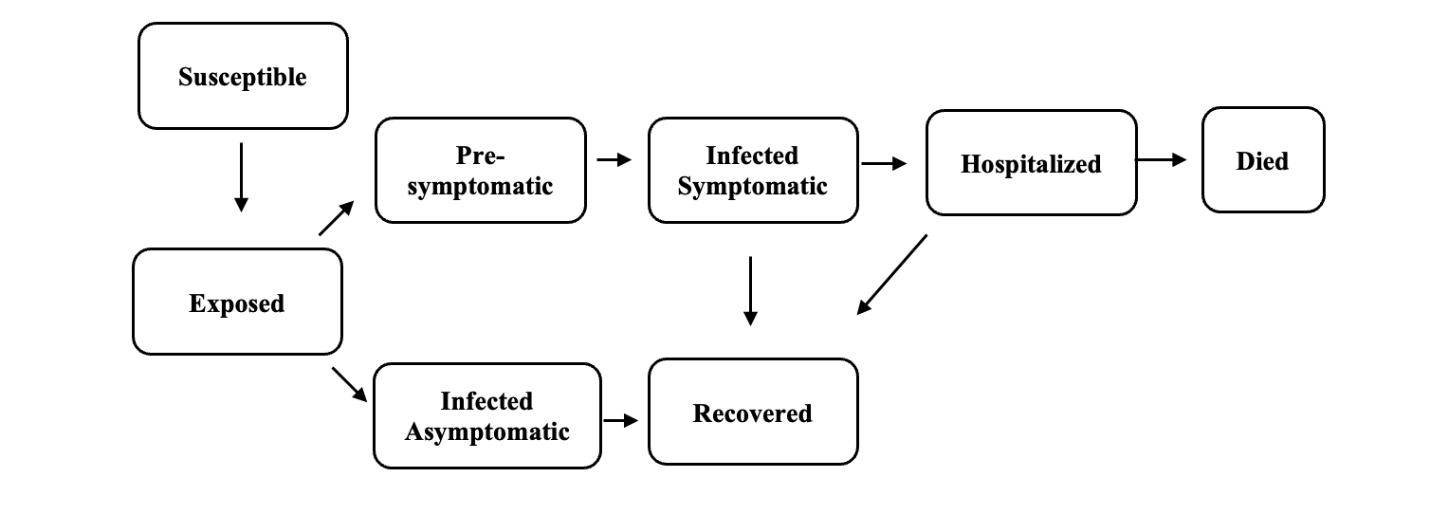


**Figure S3.** Mean number of infections in a respiratory virus model over varied numbers of iterations in the simulation.

| **Table S1. Influenza Model Inputs** | |  |
| --- | --- | --- |
| **Inputs** | **Details** | **References** |
| Population | 1,218,695 agents derived from Allegheny County 2010 census population | ^2-5^ |
| **Susceptibility-related parameters** |  |  |
| Waning of immunity from infection | 3% monthly | ^6^ |
| Vaccination strategies | 5-17 age group, 18-49 age group, 50-64 age group, 65 and up age group, random allocation, distributed at uniform rate over 6 weeks beginning September 16th |  |
| Vaccine effectiveness (VE) | Main simulations: 40% to 80% by increments of 20% | ^7^ |
| Waning of vaccine immunity | 7% monthly | ^8,9^ |
|  |  |  |
| **Influenza state durations** |  |  |
| Latent infected (E) in days | Drawn from a lognormal distribution:  μ = 1.9, σ = 1.23 | ^10^ |
| Pre-symptomatic (Ps) in days | Duration one day | ^11^ |
| Symptomatic infectious (Is) in days | Drawn from a lognormal distribution:  μ = 4, σ = 1.5 | ^12^ |
| Asymptomatic infectious (Ia) in days | Drawn from a lognormal distribution:  μ = 5, σ = 1.5 | ^12^ |
|  |  |  |
| **Simulation Parameters** |  |  |
| Simulation Period | August 15 to July 31 |  |
| Simulations Per Scenario | 100 |  |

2. *FRED transmissibility parameter*

Infectivity, typically measured for an infectious disease by R_0_ (or R_eff_ in the case of existing immunity) is not an input in FRED, which uses a combination of the transmissibility of the virus, the length of contact, and susceptibility of the recipient to determine if an infection takes place. Therefore, R_0_ and R_eff_ are outputs of our model, and the transmissibility parameter can be calibrated to produce a given R_0_ or R_eff_. Holding other parameters constant, an increase in the transmissibility parameter increases the R value.

To produce the estimations of the implied basic reproduction numbers in the table below, we ran our influenza model without vaccination or any immunity due to prior infection in Allegheny County, Pennsylvania (Table S2). The implied approximate basic reproduction number (R_0_) was calculated by taking the average of the reproductive values generated during the first week after initial case import in the simulation, as there are weekend effects in the model due to changes in interactions on weekends. R_eff_ for each of these transmissibility values would be lower in simulations in which the population is vaccinated. Most of the results in the body of the text are shown with a transmissibility value of 0.80 (~R_0_ of 1.64).

| **Table S2**: FRED transmissibility parameter and estimates of the implied R_0_ from 100 simulations without vaccination or prior immunity in Allegheny County, Pennsylvania | |
| --- | --- |
| FRED Transmissibility Parameter | Implied Basic Reproduction Number (R_0_) |
| 0.60 | 1.27 |
| 0.65 | 1.31 |
| 0.70 | 1.46 |
| 0.75 | 1.50 |
| 0.80 | 1.64 |
| 0.85 | 1.79 |
| 0.90 | 1.84 |
| 0.95 | 1.93 |
| 1.0 | 2.07 |

*3. Allegheny County Synthetic Population*

FRED synthetic populations are derived from the 2010 US Census. The FRED Allegheny County population is similar in age makeup to that of the US as a whole according to the 2020 US Census (Table S3). Particularly in a disease that transmits by the respiratory route, total infections may be impacted by local demographics, including the age distribution and household size and makeup. Our prior work showed that locations with more children and larger households will have higher attack rates but the pattern of attack rates is similar across different locations ^13^. Since Allegheny County is similar in demographics to the USA 2020 population as a whole, we feel that Allegheny County is a reasonable surrogate for the entire USA, which is computationally difficult to model using an ABM.

| **Table S3**: Age group breakdown of the Allegheny County, PA FRED population and the United States 2020 population. | | | | |
| --- | --- | --- | --- | --- |
| Age Group | FRED Allegheny County Synthetic Population Age Makeup | Number of Agents in Age Group in FRED Allegheny County Population | United States Population Age Makeup (2020) | Number of People in Age Group in the United States Population (2020)* |
| 0 to 4 | 5.17% | 63,016 | 4.39% | 14,550,623 |
| 5 to 17 | 15.30% | 186,501 | 16.23% | 53,794,218 |
| 18 to 49 | 41.49% | 505,676 | 41.91% | 138,910,394 |
| 50 to 64 | 21.05% | 256,540 | 18.68% | 61,914,726 |
| 65+ | 16.98% | 206,962 | 17.70% | 58,666,523 |

*From https://www.census.gov/popclock/data_tables.php?component=pyramid

*3. Mortality calculation method*

To determine the resulting number of deaths from each vaccination strategy, case fatality rates (CFR) from four historical outbreaks were obtained from the literature and applied to the symptomatic infection output from FRED ^14-17^(Table S4). The four outbreaks included were the US 2017-18 H3N2 seasonal influenza outbreak, the H1N1-pdm09 influenza pandemic, the 1918 influenza pandemic, and the 2020-21 wave of the COVID-19 pandemic.

|  | | | | |
| --- | --- | --- | --- | --- |
| **Table S4**: By age group case fatality rates derived from the literature for the H1N1 pdm09 flu pandemic, the 2017-18 US H3N2 seasonal outbreak, the 1918 influenza pandemic, and 2020-21 US COVID-19 pandemic. | | | | |
| Age Group | H1N1 pdm09 | 2017-18 US H3N2 | 1918 Influenza | 2020-21 US COVID-19 |
| 0 to 4 | 0.00657% | 0.00313% | 5% | 0% |
| 5 to 17 | 0.00657% | 0.00703% | 0.68% | 0.013% |
| 18 to 49 | 0.0270% | 0.0194% | 2.19% | 0.105% |
| 50 to 64 | 0.0270% | 0.0510% | 2.13% | 0.63% |
| 65+ | 0.0273% | 0.8561% | 4.5% | 6.2% |

FRED uses an accessory model to track infections by age groups, using ages commonly reported by the Centers for Disease Control and Prevention (CDC), which includes the groups: 0-4, 5-17, 18-49, 50-64, and 65+. For the 2017-18 H3N2 outbreak, the H1N1-pdm09 pandemic, and the 2020-21 wave of COVID-19, the identified literature sources used these same age groups when describing CFR, so those estimates were applied directly to the FRED symptomatic case output. For the 1918 influenza outbreak, Taubenberger et al. reported CFR using more granular age groups^15^. The 0-4 age group was described on its own and that CFR was directly applied to the FRED output for the 0-4 age group. To calculate the CFR for the other CDC age groups, the estimates for the more granular age groups in Taubenberger et al. were averaged in the following way: for 5-17 an average was taken of the reported CFRs for the 5-9, 10-14, and 15-19 age groups; for 18-49 and average was taken of the reported CFRs for the 20-24, 25-29, 30-34, 35-39, 40-44, and 45-49 age groups; for 50-64 an average was taken of the reported CFRs for the 50-54, 55-59, and 60-64 age groups; and for 65+ an average was taken of the reported CFRs for the 65-69 and 70+ age groups. These averaged CFRs were then applied to the FRED output for symptomatic infections for each of the CDC age groups.

*4. Additional results*

| **Table S5.** Total infections for selected R_0_ and vaccination strategies at 60% vaccine effectiveness with estimated mortality at rates derived from historical outbreaks, reported as mean (standard deviation). | | | | | | | |
| --- | --- | --- | --- | --- | --- | --- | --- |
| 50,000 Vaccine Doses | | | | | | | |
| ~R_0_^1^ |  | No vaccination | Vaccinate^2^  5-17 | Vaccinate  18-49 | Vaccinate  50-64 | Vaccinate  ≥65 | Random Allocation |
| 1.31 | Total Infections | 395,499 (2,804.46) | 359,020 (3,697.91) | 369,125 (3,174.47) | 375,210 (2,476.39) | 385,339 (2,890.04) | 372,285 (2,829.37) |
|  | Deaths  H1N1 pdm09 rates^3^ | 54.97  (0.37) | 50.13  (0.48) | 51.05 (0.42) | 52.20 (0.32) | 54.17 (0.38) | 51.96  (0.37) |
|  | Deaths 2017-18  H3N2 rate^4^ | 166.95  (1.01) | 146.18 (1.33) | 154.34 (1.14) | 152.67 (0.89) | 149.91 (1.04) | 153.04  (1.01) |
|  | Deaths 1918 Influenza rates^5^ | 5,620.99  (38.64) | 5,154.96 (50.95) | 5,270.63 (43.74) | 5,368.42 (34.12) | 5,512.03 (39.82) | 5,327.46 (38.98) |
|  | Deaths  COVID-19 rates^6^ | 1,224.31  (8.68) | 1,067.78 (11.45) | 1,129.64 (7.67) | 1,106.60 (7.67) | 1,099.24 (8.95) | 1,118.27 (8.76) |
| 1.5 | Total Infections | 532,727 (8,349.84) | 485,831 (2,914.99) | 499,221 (7,987.54) | 503,078 (7,344.94) | 518,115 (8,289.84) | 499,979 (7,199.70) |
|  | Deaths  H1N1 pdm09 | 79.85  (1.09) | 74.89  (0.38) | 74.94 (1.05) | 75.62 (0.96) | 78.47 (1.09) | 75.67  (0.94) |
|  | Deaths 2017-18  H3N2 rate | 290.43  (2.99) | 269.57 (1.04) | 274.31 (2.86) | 266.40 (2.63) | 257.43 (2.97) | 268.24  (2.58) |
|  | Deaths 1918 Influenza rates | 8,088.76 (115.04) | 7,570.77 (40.16) | 7,650.55 (110.05) | 7,699.51 (101.20) | 7,893.35 (114.22) | 7,668.76 (99.20) |
|  | Deaths  COVID-19 rates | 2,160.92  (21.60) | 2,007.72 (7.54) | 2,040.42 (19.00) | 1,964.03 (19.00) | 1,919.25 (21.45) | 1,992.80 (18.63) |
| 1.79 | Total Infections | 660,231 (3,957.07) | 612,558 (5,084.23) | 618,855 (3,341.82) | 620,232 (3,907.46) | 636,133 (4,198.48) | 622,651 (5,043.47) |
|  | Deaths  H1N1 pdm09 | 102.59  (0.52) | 98.76  (0.67) | 97.18 (0.44) | 97.32 (0.51) | 100.29 (0.55) | 98.37  (0.66) |
|  | Deaths 2017-18  H3N2 rate | 372.26  (1.42) | 359.48 (1.82) | 356.45 (1.20) | 341.62 (1.40) | 321.16 (1.50) | 397.63  (1.81) |
|  | Deaths 1918 Influenza rates | 10,409.67 (54.52) | 9,987.75 (70.05) | 9,919.01 (46.04) | 9,917.14 (53.84) | 10,087.15 (57.85) | 9,976.04 (69.49) |
|  | Deaths  COVID-19 rates | 3,163.58  (10.24) | 3,055.89 (13.15) | 3,024.83 (10.11) | 2,905.99 (10.11) | 2,788.45 (10.86) | 2,970.74 (13.05) |
| 1.93 | Total Infections | 735,446 (2028.82) | 716,760 (3,655.48) | 709,476 (1,702.74) | 709,176 (1,489.27) | 724,394 (1,376.35) | 7147,46 (2,072.76) |
|  | Deaths  H1N1 pdm09 | 120.24  (0.27) | 118.31 (0.48) | 114.98 (0.22) | 114.73 (0.20) | 117.46 (0.18) | 116.27  (0.27) |
|  | Deaths 2017-18  H3N2 rate | 546.53  (0.73) | 538.81 (1.31) | 528.25 (0.61) | 511.43 (0.53) | 479.23 (0.49) | 518.95  (0.74) |
|  | Deaths 1918 Influenza rates | 12,247.19 (27.95) | 12,026.64 (50.36) | 11,769.09 (23.46) | 11,729.13 (20.52) | 11,844.58 (18.96) | 11,833.74 (28.56) |
|  | Deaths  COVID-19 rates | 4,092.29  (5.25) | 4,037.97 (9.46) | 3,957.78 (3.85) | 3,806.22 (3.85) | 3,600.83 (3.56) | 3,884.68 (5.36) |
| 100,000 Vaccine Doses | | | | | | | |
| 1.31 | Total Infections | 395,499 (2,804.46) | 306,350 (14,275.91) | 353,381 (3,109.75) | 364,764 (2,845.16) | 382,940 (2,680.58) | 357,094 (2,963.88) |
|  | Deaths  H1N1 pdm09 | 54.97  (0.37) | 41.30  (1.87) | 48.07 (0.41) | 50.20 (0.37) | 53.58 (0.35) | 49.14  (0.39) |
|  | Deaths 2017-18  H3N2 rate | 166.95  (1.01) | 110.88 (5.12) | 145.93 (1.11) | 143.09 (1.02) | 136.89 (0.96) | 140.72  (1.06) |
|  | Deaths 1918 Influenza rates | 5,620.99  (38.64) | 4,288.41 (196.69) | 5,001.39 (42.85) | 5,183.87 (39.20) | 5,430.10 (36.93) | 5,052.10 (40.84) |
|  | Deaths  COVID-19 rates | 1,224.31  (7.26) | 801.81 (36.93) | 1,067.15 (7.36) | 1,027.29 (7.36) | 1,003.87 (6.93) | 1,024.39 (7.67) |
| 1.5 | Total Infections | 532,727 (8,349.84) | 463,864 (5,241.66) | 477,642 (6,925.81) | 487,815 (7,414.79) | 513,151 (7,851.21) | 478,299 (5,691.76) |
|  | Deaths  H1N1 pdm09 | 79.85  (1.09) | 69.91  (0.69) | 70.68 (0.91) | 72.39 (0.97) | 77.25 (1.03) | 71.85  (0.75) |
|  | Deaths 2017-18  H3N2 rate | 290.43  (2.99) | 238.78 (1.88) | 259.49 (2.48) | 247.93 (2.66) | 231.28 (2.81) | 249.38  (2.04) |
|  | Deaths 1918 Influenza rates | 8,088.76 (115.04) | 7,099.52 (72.22) | 7,262.34 (95.42) | 7,402.40 (102.16) | 7,727.03 (108.17) | 7,281.78 (78.42) |
|  | Deaths  COVID-19 rates | 2,160.92  (21.60) | 1,772.07 (13.56) | 1,929.79 (19.18) | 1,812.61 (19.18) | 1,727.57 (20.31) | 1,850.27 (14.72) |
| 1.79 | Total Infections | 660,231 (3,957.07) | 587,033 (1,643.69) | 596,752 (3,520.84) | 600,468 (3,602.81) | 629,571 (3,399.68) | 599,392 (49,74.95) |
|  | Deaths  H1N1 pdm09 | 102.59  (0.52) | 94.68  (0.22) | 92.74 (0.46) | 93.20 (0.47) | 98.60 (0.45) | 94.12  (0.65) |
|  | Deaths 2017-18  H3N2 rate | 372.26  (1.42) | 384.27 (0.59) | 389.00 (1.26) | 366.26 (1.29) | 332.08 (1.22) | 373.32  (1.78) |
|  | Deaths 1918 Influenza rates | 10,409.67 (54.52) | 9,562.72 (22.65) | 9,516.52 (48.51) | 9,531.30 (49.64) | 9,846.94 (46.84) | 9,538.90 (68.54) |
|  | Deaths  COVID-19 rates | 3,163.58  (10.24) | 2,877.08 (4.25) | 2,909.95 (9.32) | 2,699.83 (9.32) | 2,497.11 (8.79) | 2,786.99 (12.87) |
| 1.93 | Total Infections | 735,446 (2,028.82) | 687,933 (2,545.35) | 688,408 (1,445.66) | 688,381 (1,307.92) | 715,310 (1,359.09) | 693,685 (1,595.48) |
|  | Deaths  H1N1 pdm09 | 120.24  (0.27) | 114.82 (0.33) | 110.72 (0.19) | 110.35 (0.17) | 115.19 (0.18) | 112.23  (0.21) |
|  | Deaths 2017-18  H3N2 rate | 546.53  (0.73) | 522.04 (0.91) | 513.72 (0.52) | 483.39 (0.47) | 426.70 (0.49) | 492.45  (0.57) |
|  | Deaths 1918 Influenza rates | 12,247.19 (27.95) | 11,636.40 (35.07) | 11,383.85 (19.92) | 11,318.33 (18.02) | 11,524.13 (18.73) | 11,411.87 (21.98) |
|  | Deaths  COVID-19 rates | 4,092.29  (5.25) | 3,916.52 (6.58) | 3,850.85 (3.38) | 3,577.81 (3.38) | 3,216.84 (3.52) | 3,684.86 (4.13) |
| ^1^ ~R^0^ produced in a model with no vaccination  ^2^ All vaccinations were within the specified age group except for the random allocation scenario, in which vaccination was applied to all age groups  ^3^H1N1 pdm09 death rate by age group  ^4^ 2017-18 US H3N2 death rate by age group  ^5^1918 Influenza death rate by age group  ^6^ 2020-21 US COVID-19 death rate by age group | | | | | | | |

| **Table S6.** Total infections by age group for ~R_0_ 1.64 and vaccination strategies at 60% vaccine effectiveness with estimated mortality at rates derived from historical outbreaks, reported as mean (standard deviation). | | | | | | | |
| --- | --- | --- | --- | --- | --- | --- | --- |
|  | Age group | No vaccination | Vaccinate^1^  5-17 | Vaccinate  18-49 | Vaccinate  50-64 | Vaccinate  ≥65 | Random Allocation |
|  | 50,000 vaccines | | | | | | |
| Total Infections | 0 to 4 | 29,937 (406.04) | 28,082 (263.97) | 29,472 (506.92) | 29,772 (443.60) | 29,855 (474.69) | 28,849 (470.24) |
|  | 5 to 17 | 16,3534 (2,344.54) | 146,809 (1,233.20) | 162,908 (2,769.44) | 163,342 (2,580.80) | 163,308 (2,580.27) | 159,720 (2,667.32) |
|  | 18 to 49 | 26,2266 (2,153.86) | 25,1879 (2,140.97) | 24,2711 (2,281.48) | 256,014 (2,380.93) | 26,0956 (2,322.51) | 250,894 (2,734.74) |
|  | 50 to 64 | 102,346 (1,092.50) | 98,371 (1,200.13) | 97,757 (1,192.64) | 87,976 (862.16) | 100,533 (1,115.92) | 9,6266 (1,289.96) |
|  | 65+ | 31,663 (433.11) | 30,219 (559.05) | 30,138 (521.39) | 29,106 (430.77) | 26,846 (335.57) | 29,279 (518.24) |
|  | All ages | 589,746 (6,527.21) | 555,361 (4,887.18) | 562,987 (6,981.04) | 566,210 (6,398.17) | 581,497 (6,570.92) | 565,008 (7,458.11) |
| Deaths  H1N1 pdm09 rates | 0 to 4 | 1.48  (0.02) | 1.37  (0.01) | 1.45  (0.02) | 1.47 (0.02) | 1.47  (0.02) | 1.42  (0.02) |
|  | 5 to 17 | 8.06 (0.12) | 7.12  (0.06) | 8.03  (0.14) | 8.05 (0.13) | 8.05  (0.13) | 7.87  (0.13) |
|  | 18 to 49 | 53.11 (0.44) | 50.69  (0.43) | 49.15  (0.46) | 51.84 (0.48) | 52.84  (0.47) | 50.81  (0.55) |
|  | 50 to 64 | 20.73 (0.22) | 19.78  (0.24) | 19.80  (0.24) | 17.82 (0.17) | 20.36  (0.23) | 19.49  (0.26) |
|  | 65+ | 8.64 (0.09) | 8.18  (0.11) | 8.23  (0.11) | 7.95 (0.09) | 7.33  (0.07) | 7.99  (0.11) |
|  | Overall | 92.01 (0.86) | 87.13  (0.64) | 86.65  (0.92) | 87.12 (0.84) | 90.05  (0.86) | 87.58  (0.98) |
| Deaths 2017-18  H3N2 rate | 0 to 4 | 0.70 (0.01) | 0.65  (0.01) | 0.69  (0.01) | 0.70 (0.01) | 0.70  (0.01) | 0.68  (0.01) |
|  | 5 to 17 | 8.62 (0.12) | 7.61  (0.07) | 8.59  (0.15) | 8.61 (0.14) | 8.61  (0.14) | 8.42  (0.14) |
|  | 18 to 49 | 38.16 (0.31) | 36.42  (0.31) | 35.31  (0.33) | 37.25 (0.35) | 37.97  (0.34) | 36.51  (0.40) |
|  | 50 to 64 | 39.15 (0.42) | 37.36  (0.46) | 37.39  (0.46) | 33.65 (0.33) | 38.45  (0.43) | 36.82  (0.49) |
|  | 65+ | 271.06 (3.71) | 256.57  (4.79) | 258.01 (4.46) | 249.17 (3.69) | 229.83 (2.87) | 250.66 (4.44) |
|  | Overall | 357.70 (2.34) | 338.62  (1.75) | 340.00 (2.50) | 329.39 (2.29) | 315.56 (2.36) | 333.08 (2.67) |
| Deaths 1918 Influenza rates | 0 to 4 | 1,122.64 (15.23) | 1,042.57 (9.90) | 1,105.19 (19.01) | 1,116.46 (16.64) | 1,119.57 (17.80) | 1,081.84 (17.63) |
|  | 5 to 17 | 834.03 (11.96) | 736.42  (6.29) | 830.83 (14.12) | 833.04 (13.16) | 832.87 (13.16) | 814.57 (13.60) |
|  | 18 to 49 | 4,307.71 (35.38) | 4,111.34 (35.17) | 3,986.54 (37.47) | 4,205.03 (39.11) | 4,286.20 (38.15) | 4,120.94 (44.92) |
|  | 50 to 64 | 1,634.98 (17.45) | 1,560.32 (19.17) | 1,561.67 (19.05) | 1,405.42 (13.77) | 1,606.01 (17.83) | 1,537.84 (20.61) |
|  | 65+ | 1,424.82 (19.49) | 1,348.65 (25.16) | 1,356.22 (23.46) | 1,309.76 (19.38) | 1,208.06 (15.10) | 1,317.55 (23.32) |
|  | Overall | 9,324.18 (89.93) | 87,99.31 (67.33) | 8,840.44 (96.18) | 8869.70 (88.15) | 9,052.71 (90.53) | 8,872.75 (102.76) |
| Deaths  COVID-19 rates | 0 to 4 | 0.00 (0.00) | 0.00  (0.00) | 0.00  (0.00) | 0.00 (0.00) | 0.00  (0.00) | 0.00  (0.00) |
|  | 5 to 17 | 15.94 (0.23) | 14.08  (0.12) | 15.88  (0.25) | 15.93 (0.25) | 15.92  (0.25) | 15.57  (0.26) |
|  | 18 to 49 | 206.53 (1.70) | 197.12  (1.69) | 191.14 (1.87) | 201.61 (1.87) | 205.50 (1.83) | 197.58 (2.15) |
|  | 50 to 64 | 483.59 (5.16) | 461.50  (5.67) | 461.90 (4.07) | 415.69 (4.07) | 475.02 (5.27) | 454.86 (6.10) |
|  | 65+ | 1,963.09 (26.85) | 1,858.14 (34.66) | 1,868.57 (26.71) | 1,804.55 (26.71) | 1,664.44 (20.81) | 1,815.29 (32.13) |
|  | Overall | 2,669.15 (16.89) | 2,530.84 (12.64) | 2,537.49 (16.55) | 2,437.78 (16.55) | 2,360.89 (17.00) | 2,483.30 (19.29) |
|  | 100,000 vaccines | | | | | | |
| Total Infections | 0 to 4 | 29,937 (406.04) | 26,924 (258.47) | 29,566 (505.58) | 28,973 (495.44) | 29,909 (433.68) | 27,731 (427.06) |
|  | 5 to 17 | 163,534 (2,344.54) | 136,828 (2,982.85) | 162,702 (2,928.64) | 162,054 (2,754.92) | 163,649 (2,372.91) | 156,219 (2,437.02) |
|  | 18 to 49 | 262,266 (2,153.86) | 240,120 (19,20.96) | 249,769 (2,347.83) | 223,332 (2,255.65) | 260,140 (2,185.18) | 239,916 (2,255.21) |
|  | 50 to 64 | 102,346 (1,092.50) | 92,306 (1,532.28) | 74,558 (999.08) | 92,973 (725.19) | 98,845 (1,018.10) | 90,548 (1,023.19) |
|  | 65+ | 31,663 (433.11) | 27,838 (701.52) | 26,601 (465.52) | 28,428 (420.73) | 22,319 (216.49) | 27,216 (416.40) |
|  | All ages | 589,746 (6,527.21) | 524,017 (2,200.87) | 543,195 (7,007.22) | 535,760 (6,321.97) | 574,862 (5,863.59) | 541,631 (6,337.08) |
| Deaths  H1N1 pdm09 rates | 0 to 4 | 1.48 (0.02) | 1.33  (0.01) | 1.43  (0.02) | 1.46 (0.02) | 1.47  (0.02) | 1.42  (0.02) |
|  | 5 to 17 | 8.06 (0.12) | 6.74  (0.15) | 7.99  (0.14) | 8.02 (0.14) | 8.06  (0.12) | 7.87  (0.12) |
|  | 18 to 49 | 53.11 (0.44) | 48.62  (0.39) | 45.22  (0.48) | 50.58 (0.46) | 52.68  (0.44) | 50.81  (0.46) |
|  | 50 to 64 | 20.73 (0.22) | 18.69  (0.31) | 18.83  (0.20) | 15.10 (0.15) | 20.02  (0.21) | 19.49  (0.21) |
|  | 65+ | 8.64 (0.09) | 7.60  (0.14) | 7.76  (0.10) | 7.26 (0.09) | 6.09  (0.04) | 7.99  (0.09) |
|  | Overall | 92.01 (0.86) | 82.99  (0.29) | 81.23  (0.92) | 82.41 (0.83) | 88.33  (0.77) | 87.58  (0.83) |
| Deaths 2017-18  H3N2 rate | 0 to 4 | 0.70 (0.01) | 0.63  (0.01) | 0.68  (0.01) | 0.69 (0.01) | 0.70  (0.01) | 0.68  (0.01) |
|  | 5 to 17 | 8.62 (0.12) | 7.21  (0.16) | 8.54  (0.15) | 8.58 (0.15) | 8.63  (0.13) | 8.42  (0.13) |
|  | 18 to 49 | 38.16 (0.31) | 34.94  (0.28) | 32.49  (0.34) | 36.34 (0.33) | 37.85  (0.32) | 36.51  (0.33) |
|  | 50 to 64 | 39.15 (0.42) | 35.31  (0.59) | 35.56  (0.38) | 28.52 (0.28) | 37.81  (0.39) | 36.82  (0.39) |
|  | 65+ | 271.06 (3.71) | 238.33  (6.01) | 243.37 (3.99) | 227.73 (3.60) | 191.07 (1.85) | 250.66 (3.56) |
|  | Overall | 357.70 (2.34) | 316.42  (0.79) | 320.66 (2.51) | 301.86 (2.27) | 276.06 (2.10) | 333.08 (2.27) |
| Deaths 1918 Influenza rates | 0 to 4 | 1,122.64 (15.23) | 504.83  (9.69) | 543.24 (18.96) | 554.36 (18.58) | 560.80 (16.26) | 540.92 (16.01) |
|  | 5 to 17 | 834.03 (11.96) | 749.14 (15.21) | 887.25 (14.94) | 890.79 (14.05) | 895.98 (12.10) | 874.47 (12.43) |
|  | 18 to 49 | 4,307.71 (35.38) | 3,781.89 (31.55) | 3,517.48 (38.56) | 3,933.85 (37.05) | 4,097.20 (35.89) | 3,951.58 (37.04) |
|  | 50 to 64 | 1,634.98 (17.45) | 1,592.27 (24.48) | 1,603.78 (15.96) | 1,286.13 (11.58) | 1,705.07 (16.26) | 1,660.58 (16.35) |
|  | 65+ | 1,424.82 (19.49) | 1,308.41 (31.57) | 1,336.13 (20.95) | 1,250.24 (18.93) | 1,048.98 (9.74) | 1,376.11 (18.74) |
|  | Overall | 9,324.18 (89.93) | 7,936.54 (30.32) | 7,887.87 (96.54) | 7,915.37 (87.10) | 8,308.04 (80.79) | 8,403.66 (87.31) |
| Deaths  COVID-19 rates  COVID-19 | 0 to 4 | 0.00 (0.00) | 0.00  (0.00) | 0.00  (0.00) | 0.00 (0.00) | 0.00  (0.00) | 0.00  (0.00) |
|  | 5 to 17 | 15.94 (0.23) | 13.34  (0.29) | 15.80  (0.27) | 15.86 (0.27) | 15.96  (0.23) | 15.57  (0.24) |
|  | 18 to 49 | 206.53 (1.70) | 189.09  (1.51) | 175.87 (1.78) | 196.69 (1.78) | 204.86 (1.72) | 197.58 (1.78) |
|  | 50 to 64 | 483.59 (5.16) | 436.14  (7.24) | 439.30 (3.43) | 352.29 (3.43) | 467.04 (4.81) | 454.86 (4.83) |
|  | 65+ | 1,963.09 (26.85) | 1,725.98 (43.49) | 1,762.55 (26.09) | 1,649.25 (26.09) | 1,383.76 (13.42) | 1,815.29 (25.82) |
|  | Overall | 2,669.15 (16.89) | 2,364.56 (5.69) | 2,393.52 (16.35) | 2,214.09 (16.35) | 2,071.62 (15.17) | 2,483.30 (16.39) |
| ^1^ All vaccinations were within the specified age group except for the random allocation scenario, in which vaccination was applied to all age groups  ^2^H1N1 pdm09 death rate by age group  ^3^2017-18 US H3N2 death rate by age group  ^4^1918 Influenza death rate by age group  ^5^ 2020-21 US COVID-19 death rate by age group | | | | | | | |

*Limited vaccination*

Each age group-specific vaccination strategy was modeled with a limit of 50,000 and 100,000 vaccines, FRED transmissibility values as listed in Table S1 and VE values of 40%, 60% and 80%. (Figures S4-5, Supplementary Materials Spreadsheet).

*Mortality*

Estimated mortality for each strategy using case fatality rates from the literature are reported in Figures S6-10 and in the Supplementary Materials Spreadsheet.

*Increased Vaccination*

Infections in the increased vaccination in the 5-17 age group scenarios are reported in Figures S11-12 and in Supplementary Spreadsheet.

*4i. Individual age group vaccination strategies compared to random vaccination*

**Figure S4**: Impact of limited vaccination (50,000 or 100,000 doses) on case burden in age group specific vaccination strategies with varied ~ R_0_ and varied vaccine effectiveness (VE).


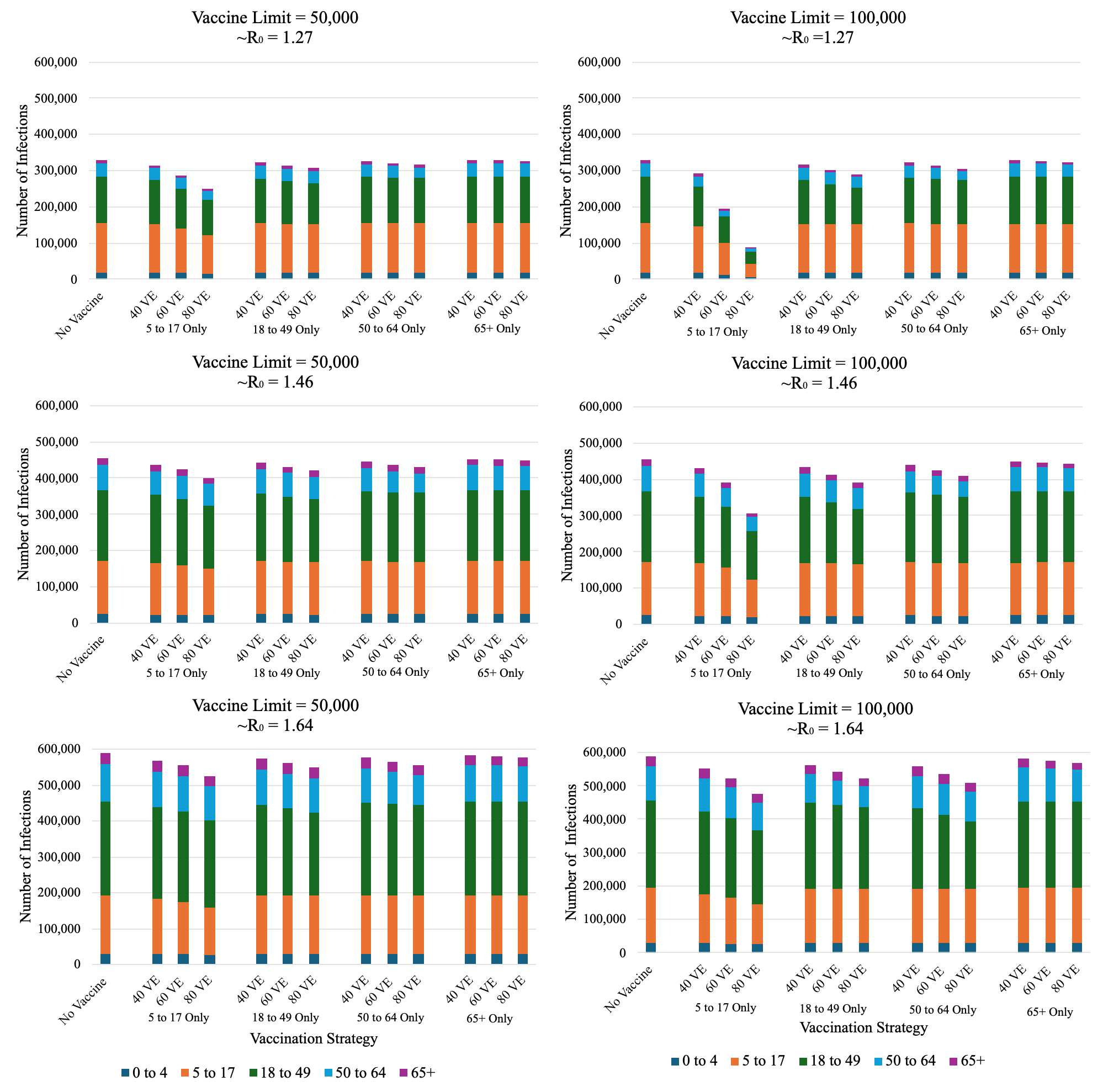


**Figure S5**: Impact of limited vaccination (50,000 or 100,000 doses) on case burden in age group specific vaccination strategies with varied ~ R_0_ and varied vaccine effectiveness (VE).


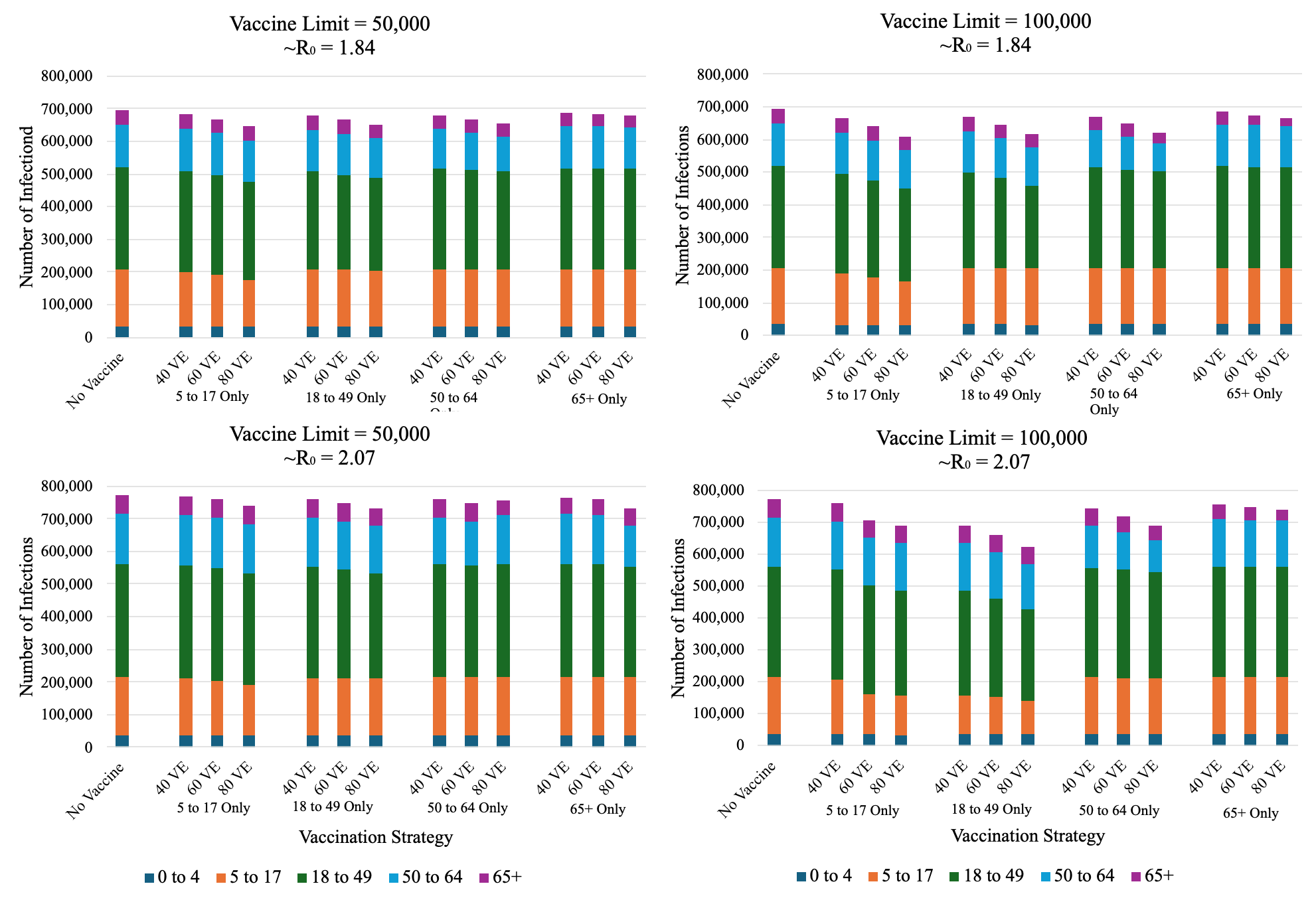


*4ii. Estimated deaths in Allegheny County population with limited vaccination by age group using reported death rates for historic pandemics and strain by varying VE.*

**Figure S6**: Impact on death burden of age specific vaccination strategies with limited vaccine supply (50,000 or 100,000 doses) and varying vaccine effectiveness (VE) for ~ R_0_ = 1.27.


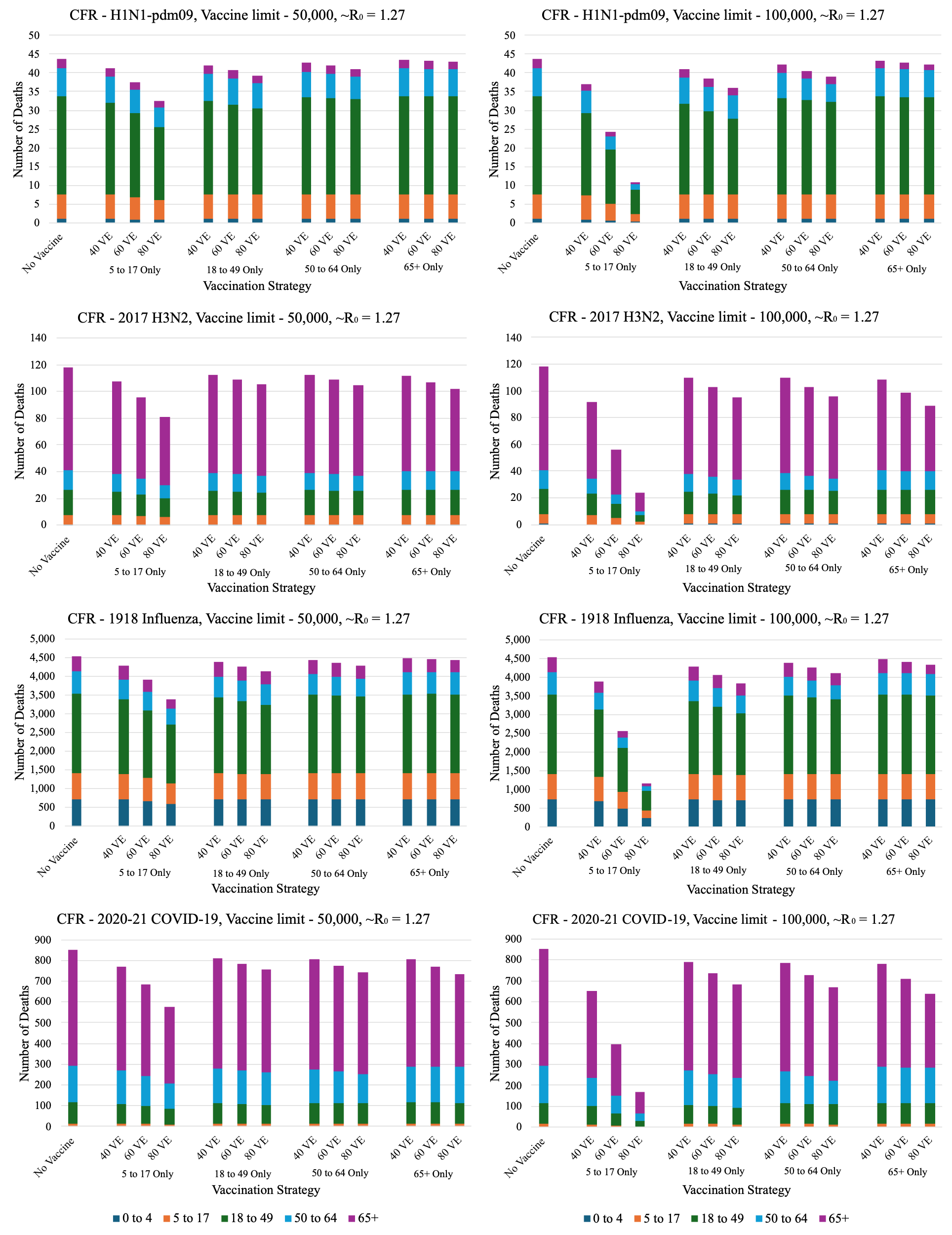


**Figure S7**: Impact on death burden of age specific vaccination strategies with limited vaccine supply (50,000 or 100,000 doses) and varying vaccine effectiveness (VE) for an ~R_0_ =1.46.

.


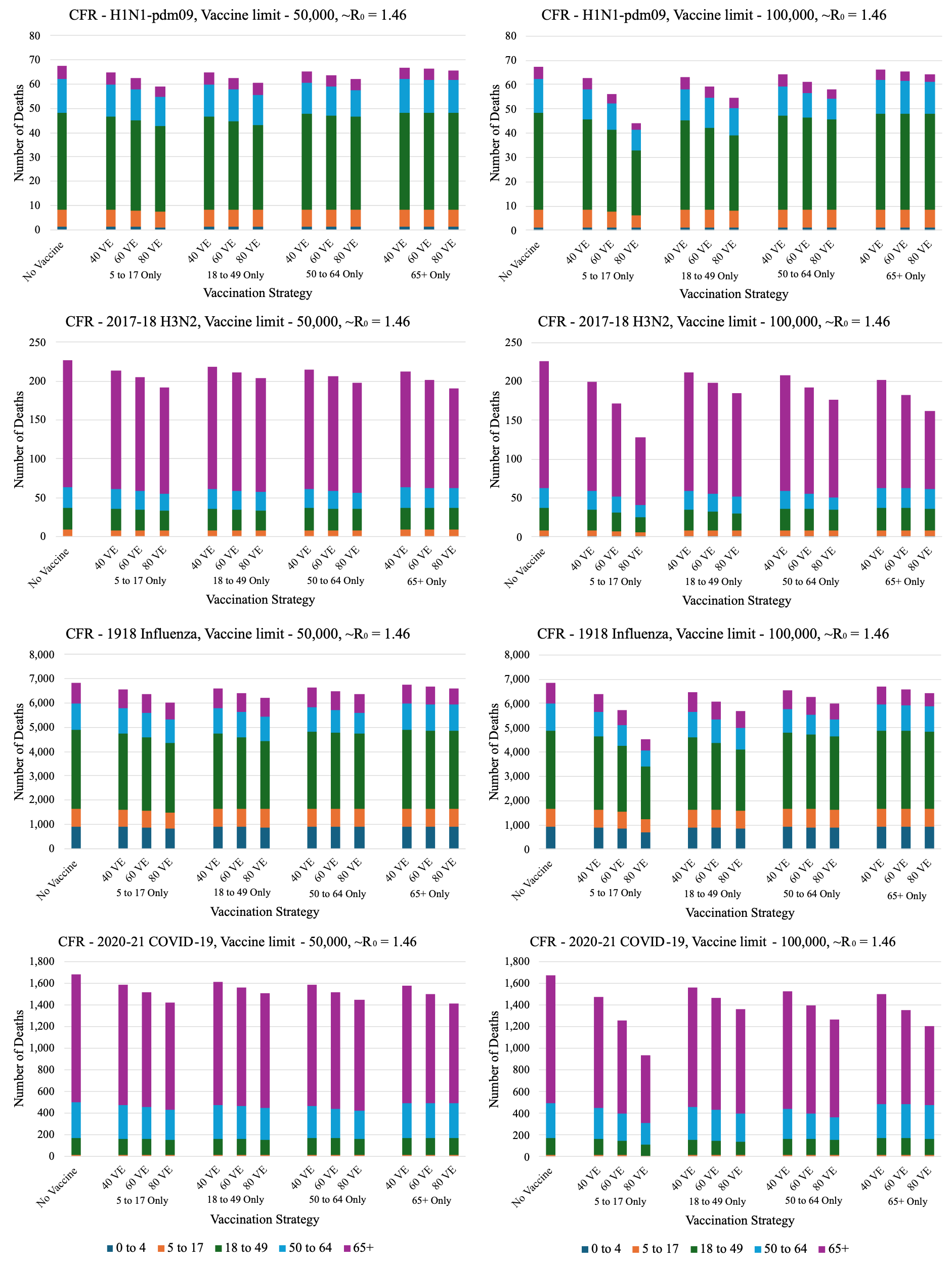


**Figure S8**: Impact on death burden of age specific vaccination strategies with limited vaccine supply (50,000 or 100,000 doses) and varying vaccine effectiveness (VE) for ~ R_0_ = 1.64.

.


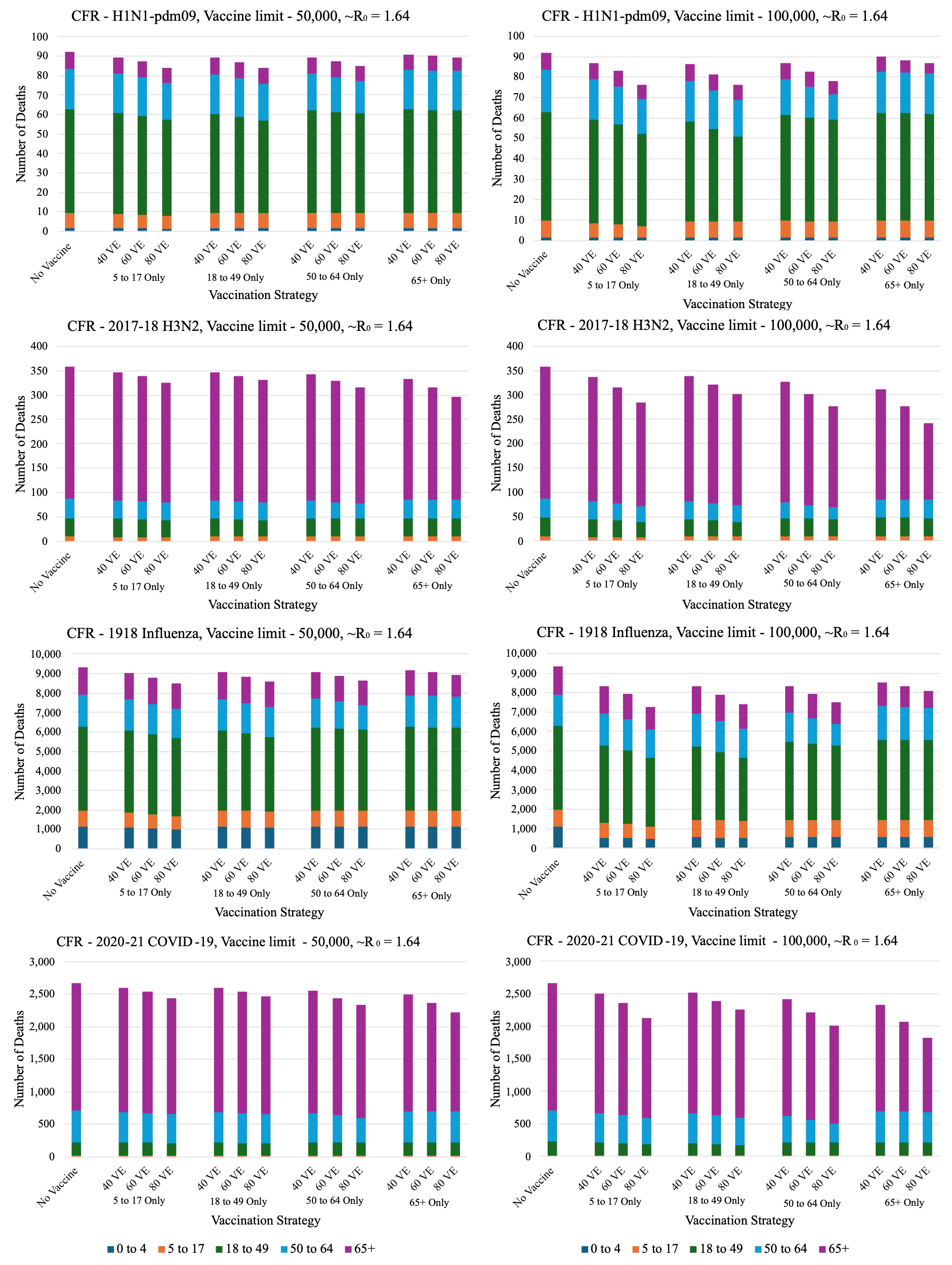


**Figure S9**: Impact on death burden of age specific vaccination strategies with limited vaccine supply (50,000 or 100,000 doses) and varying vaccine effectiveness (VE) for ~ R_0_ = 1.84.

.


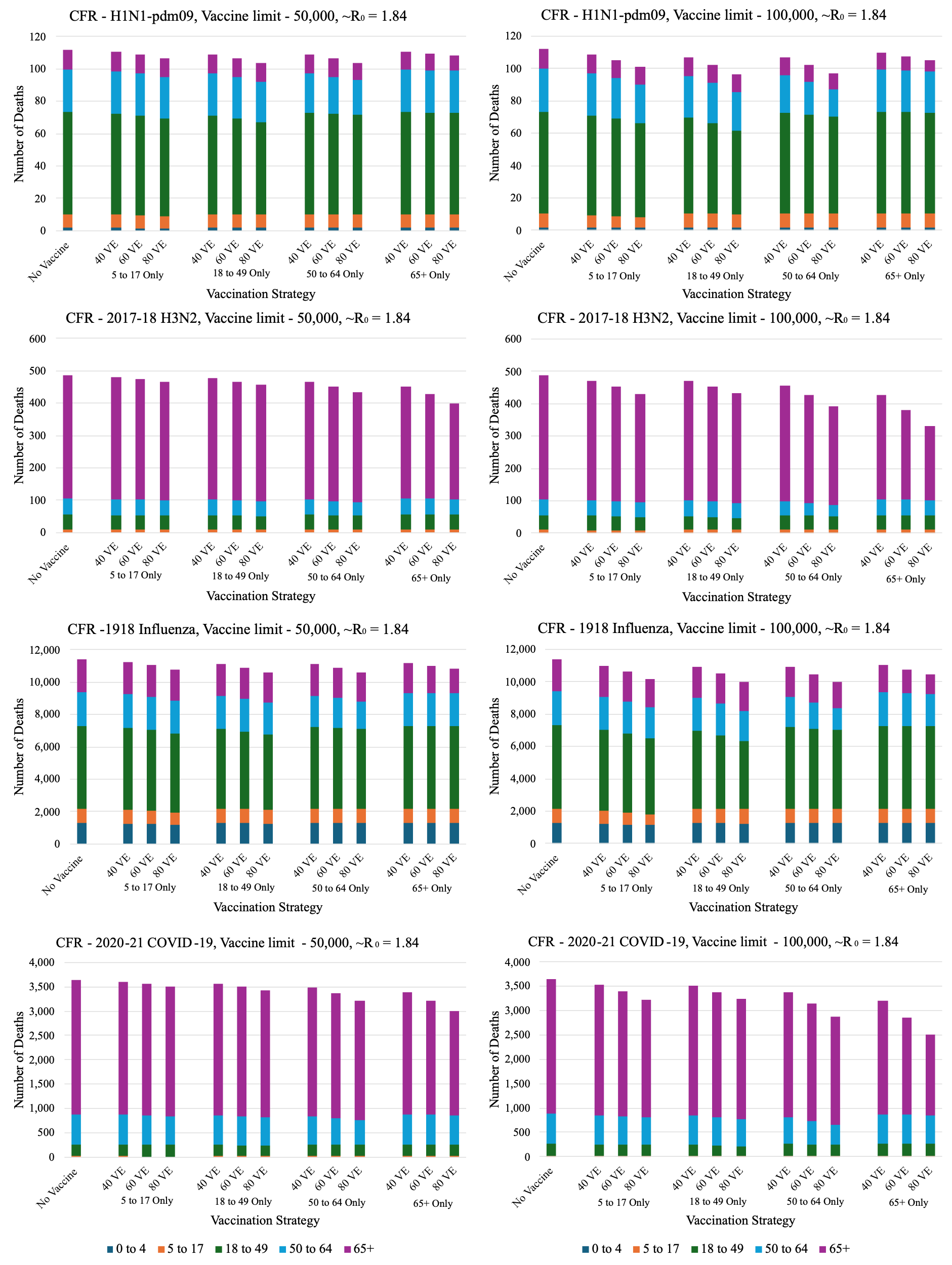


**Figure S10**: Impact on death burden of age specific vaccination strategies with limited vaccine supply (50,000 or 100,000 doses) and varying vaccine effectiveness (VE) for ~ R_0_ = 2.07.


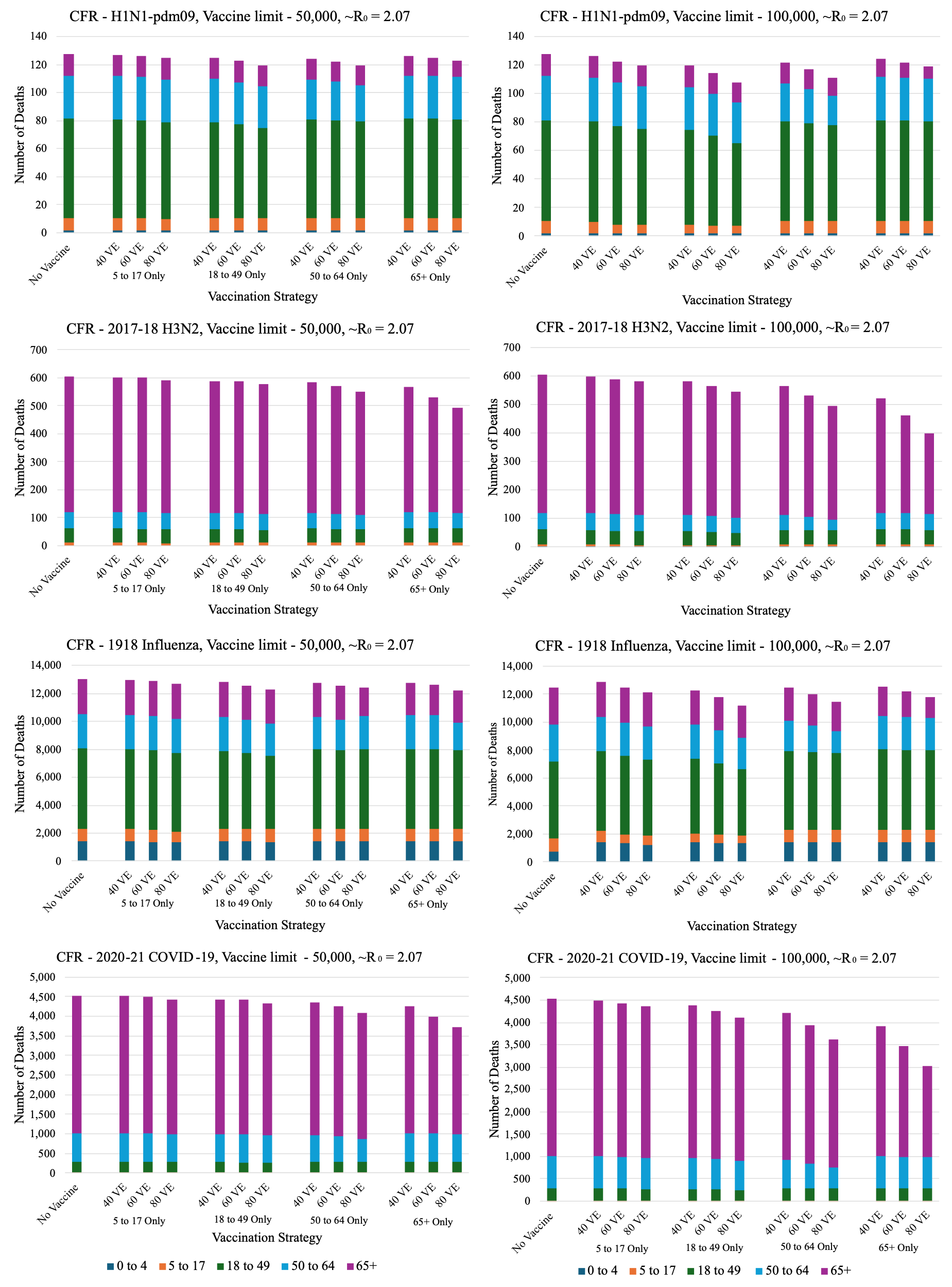


*4iii. Increasing vaccine coverage in 5-17 age group with varying (VE)*

**Figure S11**: Impact of increasing vaccination coverage in 5-17 age group on case burden by age group with varying vaccine effectiveness (VE).

.


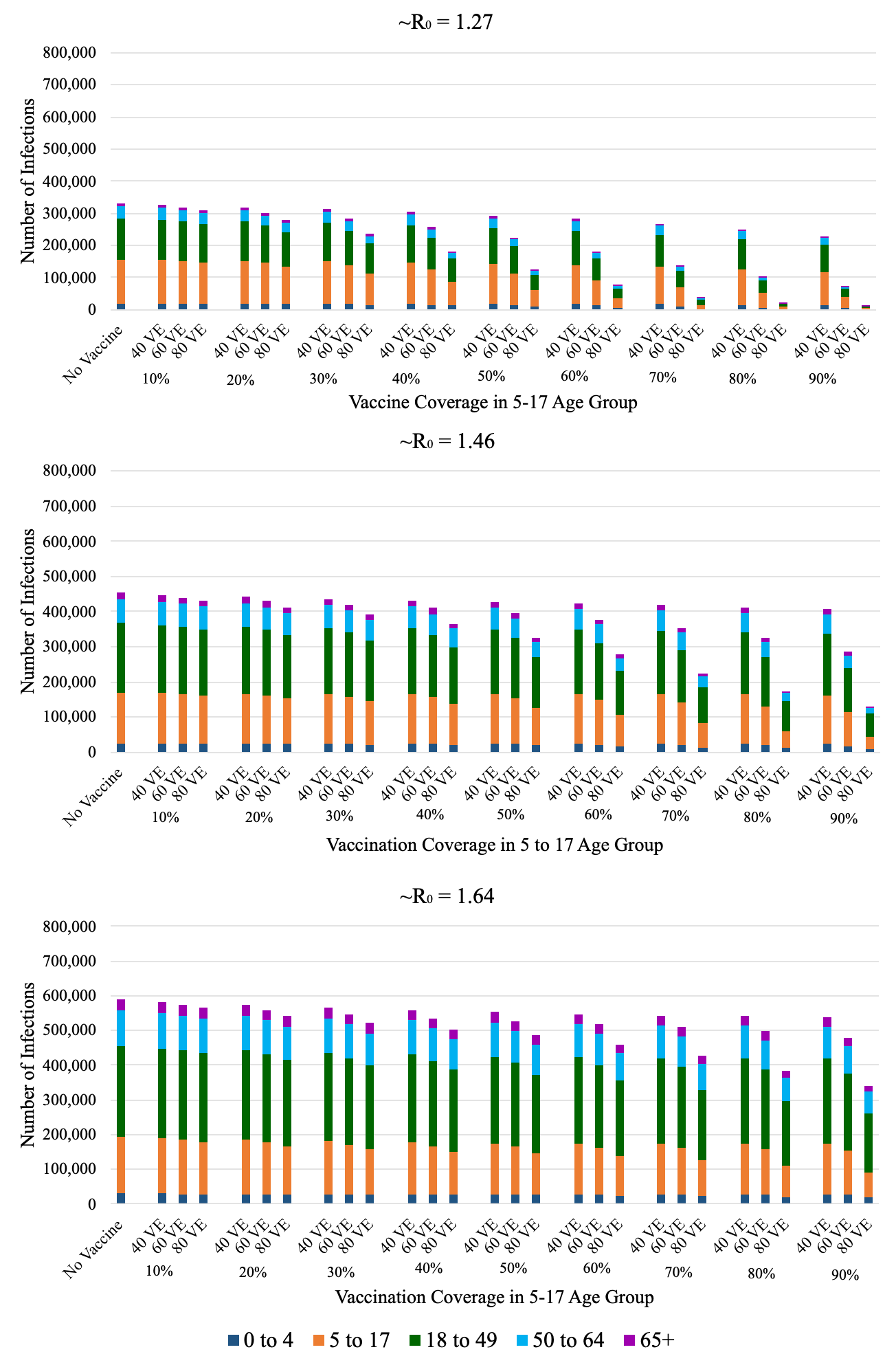


**Figure S12**: Impact of increasing vaccination coverage in 5-17 age group on case burden by age group with varying vaccine effectiveness (VE).

By va.


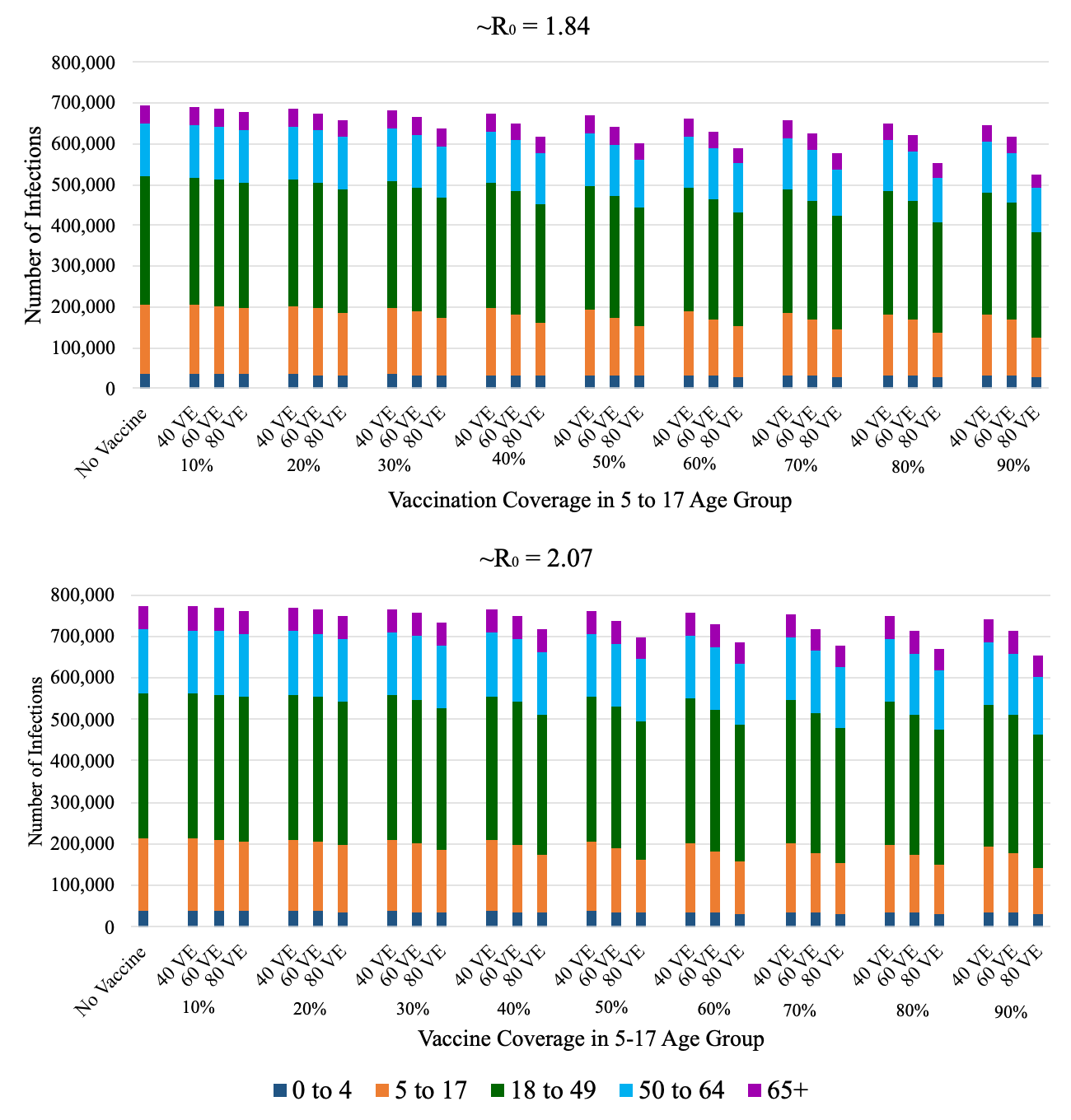


*5. References*

1. Grefenstette JJ, Brown ST, Rosenfeld R, et al. FRED (a Framework for Reconstructing Epidemic Dynamics): an open-source software system for modeling infectious diseases and control strategies using census-based populations. *BMC Public Health.* 2013;13:940.

2. Cajka JC, Cooley, P. C., & Wheaton, W. D. *Attribute assignment to a synthetic population in support of agent-based disease modeling. .* RTI Press;2010.

3. Chasteen BM, Wheaton, W. D., Cooley, P. C., Ganapathi, L., & Wagener, D. K. *Including the group quarters population in the US synthesized population database.* RTI Press;2011. RTI Press Methods Report No. MR-0020-1102

4. Chrest DP, & Wheaton, W. D. . *Using geographic information systems to define and map commuting patterns as inputs to agent-based models.* RTI Press;2009.

5. Wheaton WD, Cajka, J. C., Chasteen, B. M., Wagener, D. K., Cooley, P. C., Ganapathi, L., Roberts, D. J., & Allpress, J. L. . *Synthesized population databases: A US geospatial database for agent-based models.* RTI Press;2009.

6. Tsang TK, Perera R, Fang VJ, et al. Reconstructing antibody dynamics to estimate the risk of influenza virus infection. *Nat Commun.* 2022;13(1):1557.

7. Centers for Disease Control and Prevention. Influenza (Flu), Vaccine Effectiveness Studies. <https://www.cdc.gov/flu/vaccines-work/past-seasons-estimates.html>. Published 2024. Accessed February 8, 2024.

8. Ferdinands JM, Fry AM, Reynolds S, et al. Intraseason waning of influenza vaccine protection: Evidence from the US Influenza Vaccine Effectiveness Network, 2011-12 through 2014-15. *Clin Infect Dis.* 2017;64(5):544-550.

9. Ferdinands JM, Gaglani M, Martin ET, et al. Waning Vaccine Effectiveness Against Influenza-Associated Hospitalizations Among Adults, 2015-2016 to 2018-2019, United States Hospitalized Adult Influenza Vaccine Effectiveness Network. *Clin Infect Dis.* 2021;73(4):726-729.

10. Lessler J, Reich NG, Brookmeyer R, Perl TM, Nelson KE, Cummings DA. Incubation periods of acute respiratory viral infections: a systematic review. *Lancet Infect Dis.* 2009;9(5):291-300.

11. Centers for Disease Control and Prevention. How Flu Spreads. <https://www.cdc.gov/flu/spread/index.html>. Published 2024. Accessed November 17, 2024.

12. Carrat F, Vergu E, Ferguson NM, et al. Time lines of infection and disease in human influenza: a review of volunteer challenge studies. *Am J Epidemiol.* 2008;167(7):775-785.

13. Krauland MG, Mandell A, Roberts MS. Estimated Burden of Influenza and Direct and Indirect Benefits of Influenza Vaccination. *JAMA Netw Open.* 2025;8(7):e2521324.

14. Brazeau NF, Verity R, Jenks S, et al. Estimating the COVID-19 infection fatality ratio accounting for seroreversion using statistical modelling. *Commun Med (Lond).* 2022;2:54.

15. Centers for Disease Control and Prevention. Archived Estimated Influenza Illnesses, Medical visits, Hospitalizations, and Deaths in the United States — 2017–2018 influenza season | CDC. <https://archive.cdc.gov/www_cdc_gov/flu/about/burden/2017-2018/archive.htm>. Accessed 21 Jan 2025.

16. Shrestha SS, Swerdlow DL, Borse RH, et al. Estimating the burden of 2009 pandemic influenza A (H1N1) in the United States (April 2009-April 2010). *Clin Infect Dis.* 2011;52 Suppl 1:S75-82.

17. Taubenberger JK, Morens DM. 1918 Influenza: the mother of all pandemics. *Emerg Infect Dis.* 2006;12(1):15-22.
